# The Epidemiology of Biliary Tract Cancer and Associated Prevalence of MDM2 Amplification: A Targeted Literature Review

**DOI:** 10.1007/s11523-024-01086-5

**Published:** 2024-09-20

**Authors:** Jeremy David Kratz, Alyssa Barchet Klein, Courtney Beth Gray, Angela Märten, Hanna-Liisa Vilu, Jennifer Francesca Knight, Alexandra Kumichel, Makoto Ueno

**Affiliations:** 1 Division of Hematology, Medical Oncology and Palliative Care, Department of Medicine, University of Wisconsin School of Medicine and Public Health, University of Wisconsin, Madison, WI USA; 2 University of Wisconsin Carbone Cancer Center, Madison, WI USA; 3 William S. Middleton Memorial Veterans Hospital, Madison, WI USA; 4 Boehringer Ingelheim Pharmaceuticals, Inc., Ridgefield, CT USA; 5 Boehringer Ingelheim International GmbH, Ingelheim am Rhein, Germany; 6 Evidera Ltd, London, UK; 7 Department of Gastroenterology, Kanagawa Cancer Center, Yokohama, Japan; 8 Wi Institute Medical Research, 1111 Highland Ave Room 2784, Madison, WI 53705-2275 USA

## Abstract

Biliary tract cancer (BTC) is a rare and aggressive malignancy that is anatomically classified as gallbladder cancer (GBC), extra- and intra-hepatic cholangiocarcinoma (eCCA and iCCA) and ampullary cancer (AC). BTC is often diagnosed at an advanced stage when treatment options are limited and patients have a poor prognosis, so the identification of new drug targets is of critical importance. BTC is molecularly diverse and harbours different therapeutically actionable biomarkers, including mouse double minute 2 homolog (MDM2), which is currently being investigated as a drug target. The aim of this targeted review was to evaluate and synthesise evidence on the epidemiology of BTC and its subtypes in different geographic regions and on the frequency of MDM2 amplifications in BTC tumours. Epidemiological studies (*N* = 33) consistently demonstrated high incidence rates in South and Central Asia for BTC overall (up to 9.00/100,000) and for all subtypes, with much lower rates in Europe and the US. Among the different types of BTC, the highest global incidence was observed for CCA, mainly driven by iCCA (1.4/100,000), followed by GBC (1.2/100,000) and AC (0.18–0.93 per 100,000). Studies of MDM2 in BTC (*N* = 19) demonstrated variable frequency of MDM2 amplification according to subtype, with consistently high MDM2 amplification rates in GBC (up to 17.5%), and lower rates in CCA (up to 4.4%). The results from this literature review highlight the geographic heterogeneity of BTC and the need for standardised clinicopathologic assessment and reporting to allow cross-study comparisons.

**Supplementary Information:**

The online version contains supplementary material available at 10.1007/s11523-024-01086-5.

## Key Points

**Table:** 

The incidence of biliary tract cancer (BTC) varies considerably according to geography and anatomical subtype, with highest rates observed in South and Central Asia and cholangiocarcinoma being the most common subtype.
MDM2 represents one of several novel drug targets for the treatment of BTC.
The prevalence of MDM2 amplification varies according to the anatomical subtypes of BTC, with the highest amplification rates in gallbladder cancer (up to 17.5%).

## Introduction

**Fig. 1:**
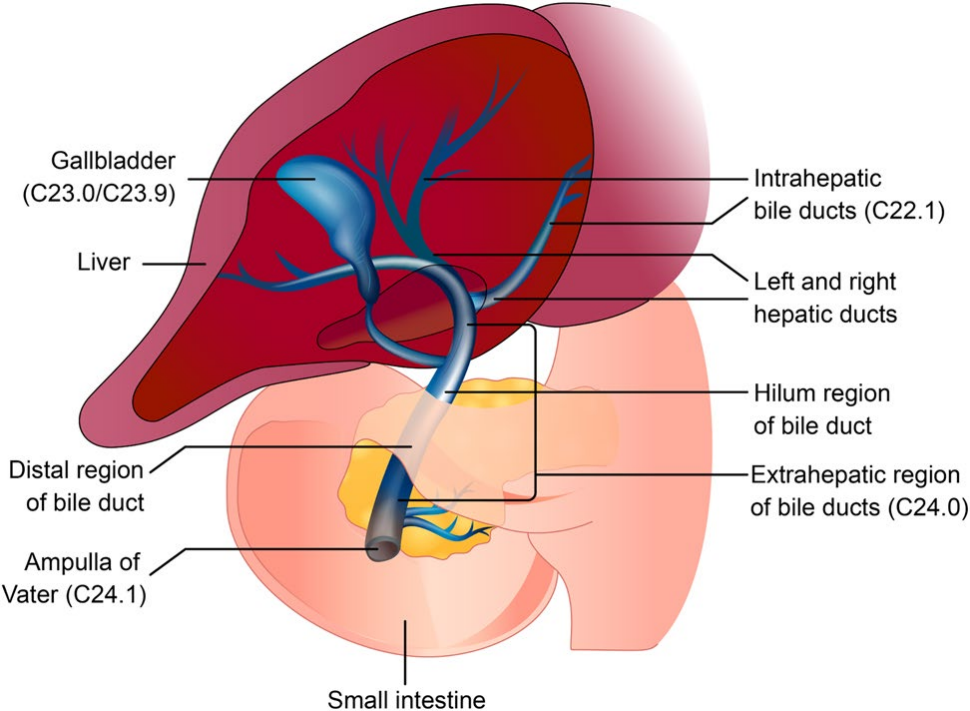
Regions of the biliary tract. Primary tumour sites described in this literature review involve the gallbladder (GBC [ICD-10 codes C23.0/C23.9]), intrahepatic bile ducts (iCCA [ICD-10 code C22.1]), extrahepatic bile ducts (eCCA [ICD-10 code C24.0] and ampullary cancer [ICD-10 code C24.1]). Additional ICD-10 site codes for BTC include ‘overlapping lesion of biliary tract’ (C24.8) and ‘biliary tract, unspecified’ (C24.9). ICD-10 codes are as published by the World Health Organization [2]. *BTC* biliary tract cancer, *eCCA* extrahepatic cholangiocarcinoma, *iCCA* intrahepatic cholangiocarcinoma, *ICD* International Classification of Disease

Biliary tract cancer (BTC) comprises a heterogeneous group of aggressive epithelial malignancies of the gallbladder and biliary tract, anatomically classified as gallbladder cancer (GBC), extra- and intra-hepatic cholangiocarcinoma (eCCA and iCCA) and ampulla of Vater cancer, also known as ampullary cancer (AC; Fig. 1) [1].

While BTC accounts for <1% of all human cancers, it is the second most common primary hepatobiliary malignancy following hepatocellular carcinoma [3]. Risk factors for BTC are wide-ranging and vary across the different histological subtypes. For CCA, the greatest risk factors include choledochal cysts (odds ratios [ORs] for iCCA and eCCA, respectively: 26.7 and 34.9), cirrhosis (ORs: 15.3 and 3.8), and bile duct stones (ORs: 10.1 and 18.6) [4]. In South-East Asian countries like Thailand, liver fluke infection is a prominent risk factor for CCA, increasing the risk by five- to six-fold [5]. GBC is associated with risk factors such as irritation or inflammation of the gallbladder, including the presence of gallstones (OR: 23.8), which are found in up to 84% of GBC patients [6]. Additionally, chronic *Salmonella Typhi* carrier state can increase the risk of GBC by four-fold [7]. The risk factors for AC have been less extensively studied; however, conditions such as diabetes mellitus, cholecystolithiasis (gallstones), and chronic pancreatitis have been associated with increased risk (ORs: 4.8, 14.1 and 8.9, respectively) [8]. There are no routine surveillance screenings for BTC in high-risk populations [3, 9]. Furthermore, as the symptoms of BTC are often non-specific, the disease is usually diagnosed at an advanced stage when treatment options are limited [1, 10]. Patients with BTC therefore have a poor prognosis, with 5-year survival rates of ~15% reported in the US [11, 12].

In addition to distinct anatomic and histologic subtypes, BTC is molecularly diverse, displaying differences in gene expression, tumour mutational burden, copy number alterations, gene fusions and translocations [13–15]. Genomic alterations vary in frequency among the anatomical subtypes and include mutations (e.g., *TP53*, *IDH1*, *BRAF* and *KRAS*), amplifications (e.g., *ERBB2*, *MDM2*, *CCNE1* and *MYC*), and gene fusions (e.g., those involving *FGFR2*) [15–17]. Importantly, the molecular characterisation of BTC has the potential to benefit patients by facilitating the identification of prognostic, predictive and therapeutically actionable biomarkers [10, 14, 15]. Actionable biomarkers have been detected in 15.0–39.1% of BTC samples, with frequency varying according to primary site [16–18].

While other genomic alterations in BTC are well established in the literature and have been covered in previously published reviews, there is a paucity of synthesised evidence on mouse double minute 2 homolog (MDM2) amplification in BTC [19–21]. As a primary negative regulator of the tumour suppressor p53, MDM2 is regarded as an oncoprotein with a key role in the regulation of cell growth and apoptosis [22, 23]. Due to its role in p53 suppression, MDM2 represents a promising novel drug target for the treatment of several cancers, including BTC. MDM2 amplification promotes p53 depletion, leading to tumour cell proliferation, and has been detected in approximately 3.5–7.0% of all cancers [23–27]. However, there is considerable variation in the frequency of MDM2 amplification across different types of malignancies [25–27]. Brigimadlin (an MDM2-p53 antagonist) is currently under investigation for the treatment of BTC in the phase II Brightline-2 trial (ClinicalTrials.gov identifier: NCT05512377) [27]. Other inhibitors of MDM2 are being evaluated in basket trials; these include milademetan in the phase II MANTRA-2 trial (NCT05012397), in which 8/39 patients (20.5%) have BTC, ASTX295 in a phase I/II trial of solid tumour types and KT-253 in a phase I trial of assorted malignancies including solid tumours (proportion of patients with BTC not reported) [28–31].

There are few detailed epidemiological studies of BTC, and interpretation of such data is complex for a variety of reasons. Understanding the epidemiology requires dedicated pathology and radiographic assessments to define the primary site of origin in BTC. Inaccuracies in estimating incidence and prevalence may also arise out of pooling of data for liver cancer and BTC, leading to the inclusion of hepatocellular carcinoma or other rare cancer types [3]. Inconsistent reporting of the topography and morphology codes used to distinguish histologic subtypes of BTC can further confound the results [3, 10]. Tracking changes in BTC incidence over time may also be impacted by updates in International Classification of Disease (ICD) codes [1].

The primary aim of this targeted review was to provide both a qualitative synthesis of the evidence on the incidence and prevalence of BTC and its subtypes in different geographic regions, as well as to clarify knowledge on the frequency of MDM2 amplification in BTC tumours.

## Materials and Methods

Two targeted literature reviews were conducted to identify evidence on (1) the incidence and prevalence of BTC overall and by primary site, and (2) the proportion of BTC with MDM2 amplification.

### Literature Searches

Searches were conducted in databases including Medline and Embase and in the proceedings of international scientific meetings including the American Society of Clinical Oncology (ASCO), the European Society for Medical Oncology (ESMO) and the Japanese Society for Medical Oncology (JSMO). Searches included studies in humans published up to 11 September 2023. For the review of the epidemiology of BTC, the searches were limited to literature published after 1 January 2020. The applied search strategies were developed by a specialised librarian using a combination of indexing terms (Medical Subject Headings terms in MEDLINE and Emtree terms in Embase), as well as free-text and controlled vocabulary terms specific to each database (Tables S11 and S12 in the electronic supplementary material [ESM]). The search strategy for the epidemiology of BTC included retrospective and prospective observational studies, while the search strategy for the proportion of BTC with MDM2 amplification had no restrictions on study design. No language or geographical restrictions were applied to the searches before screening. In addition, epidemiological and genetic databases including Surveillance, Epidemiology, and End Results (SEER), International Agency for Cancer Research (IARC) via the Global Cancer Observatory (Globocan) and MyCancerGenome were consulted for data extraction.

### Study Selection

**Fig. 2:**
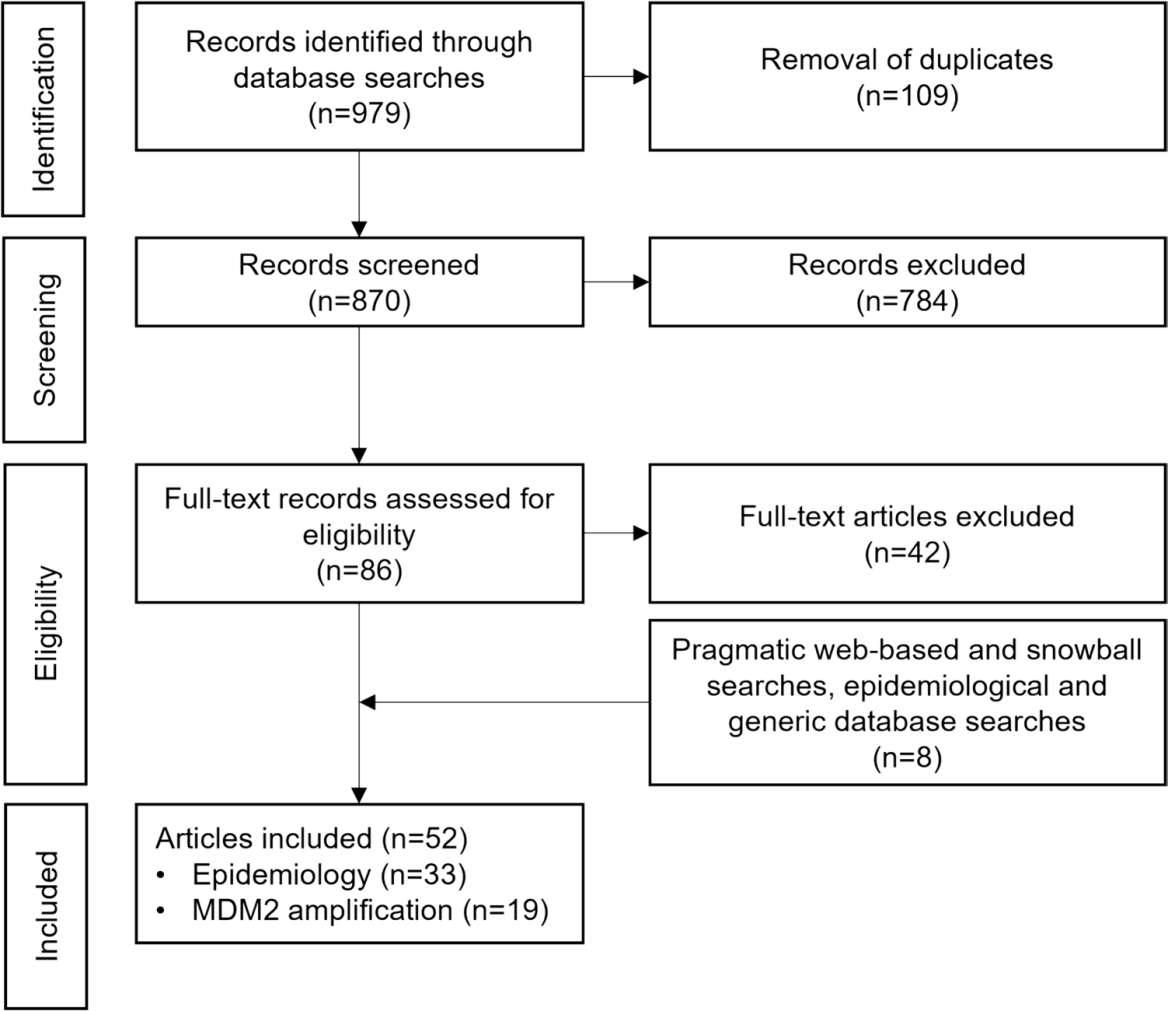
PRISMA flow diagram of literature identification and selection process. *MDM2* mouse double minute 2 homolog, *PRISMA* Preferred Reporting Items for Systematic Reviews and Meta-Analyses

Titles and abstracts were manually screened by one researcher, and those which appeared relevant were screened at full-text level. Studies published in the English language containing epidemiological data on the prevalence and incidence of BTC and/or anatomical subtypes of BTC, in the following countries and regions of interest, were included: the US, the EU-4 (France, Germany, Spain, Italy), the UK, Nordic countries (Sweden, Norway, Finland, Denmark) and Asia (China, Japan, South Korea, Taiwan and Thailand). Articles not adhering to these criteria were excluded from further consideration; additional reasons for exclusion included irrelevant study design (non-epidemiological studies including incidental diagnoses, or case studies) and studies focused on hepatocellular carcinoma. For MDM2 amplification, articles published in English providing data on the frequency of MDM2 gene amplification in BTC and/or anatomical subtypes of BTC were included, regardless of the study country and design. Articles selected for inclusion were validated by a second reviewer and discrepancies were resolved by consensus. The process of including and excluding studies has been summarised in Fig. 2. Data from full-text articles were retrieved using a data extraction form and a comprehensive quality check was performed to validate the extracted data.

## Results

### Literature Search

The literature search identified 870 individual publications in selected literature databases and conference proceedings. Of these, 784 were excluded after screening and a further 42 were excluded after full-text analysis. Five additional publications were identified through pragmatic web-based searches and snowballing searches based on reference lists of other relevant publications. Additional data were extracted from the SEER, Globocan, and MyCancerGenome databases. A total of 52 sources were included in the qualitative analysis, of which 33 (63.5%) and 19 (36.5%) contained data on BTC epidemiology and MDM2 amplification, respectively. The flowchart of the study selection is shown in Fig. 2. A summary of the included publications is shown in Table S1 and Table S2 (see ESM).

### Incidence

In 2019, the global age-standardised rate (ASR) of incidence for BTC (cases per 100,000 persons) was 2.5, based on data collected as part of the Global Burden of Disease Study [32]. However, there is considerable variation in reported incidence according to primary site, geographic region and sex [33–35]. Incidence data for BTC among countries of interest to this review are summarised in Table 1; global incidence ranges inclusive of other countries are presented in Table S3 (see ESM).

**Table 1:** Age-standardised incidence rates of BTC by geographic region of interest [12, 33, 34, 38–45]

Site	Study	Data year (s)	Incidence (ASR/100,000)		
			Asia^a^	Europe^a^	US
BTC	Baria 2022 [40]	2008–2012	3.26 (CN) to 9.00 (KR)	1.99 (UK) to 3.59 (IT)	2.32
	Koshiol 2022 [12]	2001–2015			5.04
	Chen 2022 [41]	2019	2.01 (CN)		
iCCA	Florio 2020 [33]	2008–2012	0.63 (CN) to 2.80 (KR)	0.45 (NO) to 1.15 (UK)	0.78
	Baria 2022 [40]	2008–2012	0.59 (CN) to 2.18 (KR)	0.68 (DE) to 1.01 (FR)	0.64
	Rumgay 2022 [34]	2018	0.6 (JP) to 5.2 (TH)	0.9 (NO) to 2.2 (UK)	1.1
	Koshiol 2022 [12]	2001–2015			1.17
	Tella 2023 [42]	2000–2017			1.07
	Javle 2022 [43]	2001–2017			1.19
	Hong 2022 [44]	1999–2019	2.7 (Korea)		
eCCA	Florio 2020 [33]	2008–2012	0.55 (CN) to 2.24 (KR)	0.29 (UK) to 0.74 (DE)	0.58
	Baria 2022 [40]	2008–2012	0.93 (CN) to 2.71 (KR)	0.34 (UK) to 0.87 (IT)	0.57
	Koshiol 2022 [12]	2001–2015			1.25
	Tella 2023 [42]	2000–2017			0.74
	Javle 2022 [43]	2001–2017			2.46
	Kang 2022 [38]	2019	4.1 (KR)		
GBC	GLOBOCAN [39]	2020	1.2 (CN) to 2.9 (KR)	0.39 (NO/FR) to 0.89 (SE)	0.68
	SEER-22 [45]	2020			1.1
	Kang 2022 [38]	2019	2.4 (Korea)		
AC	Baria 2022 [40]	2008–2012	0.23 (CN) to 0.93 (KR)	0.34 (UK) to 0.45 (ES)	0.32
	Koshiol 2022 [12]	2001–2015			0.76

*AC* ampullary cancer, *ASR* age-standardised rate, *BTC* biliary tract cancer, *CN* China, *DE* Germany, *eCCA* extrahepatic cholangiocarcinoma, *ES* Spain, *FR* France, *GBC* gallbladder cancer, *iCCA* intrahepatic cholangiocarcinoma, *IT* Italy, *JP* Japan, *KR* South Korea, *NO* Norway, *SE* Sweden, *TH* Thailand, *UK* United Kingdom, *US* United States

^a^It should be noted that ranges reported for Asia and Europe are those for specific countries of interest to this review; Asian countries were China, Japan, South Korea, Taiwan and Thailand. European ranges reflect data for France, Germany, Spain, Italy, the UK, Sweden, Norway, Finland and Denmark

#### Global Incidence by Primary Site

Among the different types of BTC, the combined incidence of iCCA and eCCA is highest, followed by GBC and AC [12, 36–38]. The incidence of CCA is mainly driven by iCCA; during 2008 to 2012, the global annualised ASR ranged from 0.26 to 2.80 for iCCA, compared with 0.08 to 2.24 for eCCA during the same time period (Table S3, see ESM) [33]. More recent global data for iCCA in 2018 showed a global incidence rate of 1.4 (Table S3) [34]. No global data published since 2012 were identified for eCCA, reflecting the smaller volume of data available compared with iCCA.

Global incidence data for GBC were available from one source, which reported an ASR of 1.2 for 2020 (ranging from 0.03 to 8.5; Table S3, see ESM) [39]. Similarly, data on AC are generally scarce, with one international study reporting data for 22 countries (2008–2012) showing that AC has the lowest incidence rate of all types of BTC, with ASRs ranging from 0.18 to 0.93 (Table S3) [40].

#### Incidence by Geographic Location

Geographically, the incidence of BTC varies widely (Table S3, see ESM). Across the geographic regions focused on in this review, locations with the highest incidence of BTC were South and Central Asia, where BTC was estimated to account for 2.9% of all new cancer diagnoses in 2018 [35].

Two studies using global data from 2008 to 2012, available from IARC, showed that, among the countries focused on in this review, the highest incidence rates of iCCA (2.80 and 2.18) and eCCA (2.24 and 2.71) occurred in South Korea (Table 1) [33, 40]. Other countries in Asia, Europe and the US had considerably lower rates in these studies (Table 1) [33].

Beyond this, there is a paucity of recent studies comparing the incidence of eCCA by geography and few individual studies describe country-specific data. Two studies from Asia reported an ASR of eCCA of 4.1 in South Korea (2019; Table 1) and 5.1/2.2 (male/female) in Japan (2016–2017; Table S6, see ESM) [38, 46]. Two studies from Europe report an ASR of eCCA of 0.94 in France (2018–2019) and 0.4/0.3 (male/female) in Finland (2013–2017; Table S5, see ESM) [37, 47]. Three US-based studies reported incidence rates of eCCA for different time periods, with considerable variation: 2.46 (2001–2017), 1.25 (2001–2015) and 0.74 (2000–2017; Table 1) [12, 42, 43]. The inconsistent incidence rates across studies may be due to differences in assessment dates, the population used for age standardisation, and the ICD-10/ICD-Oncology (O)-3 site and morphology codes used for data extraction (Table S2, see ESM).

In contrast, several articles describe the incidence of iCCA by geography, including a global study that published the ASR of iCCA incidence for 2018 by country (Table 1; Table S3, see ESM) [34]. The authors showed a high incidence rate of iCCA in Asian countries (Thailand [5.2], South Korea [3.3] and China [2.1]; Table 1, Table S3) [34]. Estimates of the ASR of iCCA incidence in European countries in 2018 ranged from 0.9 in Norway to 2.2 in the UK, and the ASR of iCCA in the US was 1.1 (Table 1; Table S3) [34]. For reasons that are unclear, estimates for the ASR of iCCA incidence varied somewhat across different US-based studies, which reported rates from 0.64 (2008–2012) to 1.19 (2001–2017) for iCCA (Table 1) [12, 40, 42, 43].

For GBC, incidence rates reported for 2020 varied greatly among countries (Table S3, see ESM), ranging from 0.39 to 2.9 among countries focused on in this review (Table 1) [39]. Incidence rates were relatively high in Asia (ranging from 1.2 in China to 2.9 in South Korea), compared with European countries (ranging from 0.39 in Norway and France, to 0.89 in Sweden), and the US (at 0.68) (Table 1) [39]. However, US-specific data for the same year (2020) available through the SEER database showed a higher ASR rate of 1.1 (Table 1) [45].

Incidence rates of AC reported by one study (2008–2012) were generally low across all regions of interest, with the highest rates reported in Asia (ranging from 0.23 in China to 0.93 in South Korea), followed by European countries (from 0.34 in the UK to 0.45 in Spain), and the US with 0.32 (Table 1) [40].

#### Incidence by Sex

Data for BTC overall suggest a slightly higher incidence in females (2.6) compared with males (2.4), but this trend varies by primary site (Table S3, see ESM) [32]. Incidence rates of CCA, for example, were typically higher among males than females in the US, Asia, the EU-4, the UK and in Nordic countries (Tables S3–S6, see ESM) [12, 34, 35, 43, 46–50]. Conversely, the incidence of GBC was generally higher in females than in males in most countries (Tables S3–S6) [12, 35, 39, 51]. Exceptions included Thailand and South Korea, where there was a higher incidence of GBC in males compared with females, and Japan where GBC incidence was similar between males and females (Table S6) [39, 46, 50]. Data from a global study, as well as individual studies from the US and Japan (Tables S3, S4, S6, see ESM), showed higher incidence of AC among males than females [12, 35, 40, 46].

#### Trends in Incidence

Studies reporting changes in the incidence of BTC over time were generally focused on specific primary sites, with the majority of studies reporting on either CCA or GBC. A global study by Florio et al. reported an increase in incidence of both iCCA and eCCA over time (1993–2012) (Table 2) [33]. Furthermore, although taken from separate studies, estimates of iCCA incidence in 2018 were generally higher than those reported for 2008–2012, suggesting a continued increase (Table S3, see ESM) [33, 34]. Among the countries focused on in this report, the largest annual percentage change (APC) in iCCA incidence rate between 1993 and 2012 occurred in China (11.1%), followed by Germany (7.5%) and France (6.5%) (Table 2; Table S7, see ESM) [33]. An increase in the incidence of iCCA is also documented by several US-based studies, which report an APC ranging from 2.0% (1993–2012) to 9.3% (2013–2017) depending on the study period and definitions used (Table S7) [12, 33, 43, 52–54]. Using data for 1993 to 2012, only a few countries, including Japan, Thailand and Denmark, showed a slight decrease in the incidence of iCCA (Table S7) [33].

**Table 2:** Temporal trends in the age-standardised incidence of BTC by geographic region of interest [12, 33, 36–38, 43–45]

Site	Study	Data year(s)	Incidence trends (APC in ASR/100,000, %)		
			Asia	Europe	US
BTC	Koshiol 2022 [12]	2001–2015			+1.76
	Rahman 2022 [36]	1993–2019		−0.13 (SE)	
iCCA	Florio 2020 [33]	1993–2012	−1.0 (TH); +11.1 (CN)	−1.0 (DK); +7.5 (DE)	+2.0
	Rahman 2022 [36]	1993–2019		+1.7 (SE)	
	Koshiol 2022 [12]	2001–2015			+6.7
	Javle 2022 [43]	2013–2017			+9.3
	Hong 2022 [44]	2008–2019	−4.38 (KR)		
eCCA	Florio 2020 [33]	1993–2012	+1.4 (JP); +8.8 (TH)	+2.0 (FR); +4.0 (IT)	+2.4
	Koshiol 2022 [12]	2001–2015			+0.72
	Javle 2022 [43]	2013–2017			−1.97
	Kang 2022 [38]	1999–2019	+0.76 (KR)		
GBC	SEER-22 [45]	2015–2019			−0.1
	Koshiol 2022 [12]	2001–2015			−0.25
	Rahman 2022 [36]	1993–2019		−2.82 (SE)	
	Kang 2022 [38]	1999–2019	−0.87 (KR)		
AC	Koshiol 2022 [12]	2001–2015			Male: −0.14 Female: −0.08
	Ghiringhelli 2023 [37]	2015–2019		Male: −14.6 (FR) Female: −16.6 (FR)	

*AC* ampullary cancer, *APC* annual percentage change, *ASR* age-standardised rate, *BTC* biliary tract cancer, *CN* China, *DE* Germany, *DK* Denmark, *eCCA* extrahepatic cholangiocarcinoma, *FR* France, *GBC* gallbladder cancer, *iCCA* intrahepatic cholangiocarcinoma, *IT* Italy, *JP* Japan, *KR* South Korea, *SE* Sweden, *TH* Thailand, *US* United States

In general, the global study by Florio et al. recorded relatively small increases in the incidence of eCCA between 1993 and 2012 (Table 2, Table S7, see ESM) [33]. Exceptions included Thailand, with an APC of 8.8% and Italy, with an APC of 4.0% (Table 2); results for the US showed an APC of 2.4% for eCCA [33]. However, more recent data (2013–2017) showing incidence trends for eCCA in the US indicate a minimal negative APC of −2.0% (Table 2) [43].

Data on GBC incidence by sex from the Globocan database show that incidence trends vary between countries (Table S7, see ESM) [39]. Based on the most recent 5-year period available, large decreases in GBC incidence in the male population were observed in Norway (−5.5%) and China (−2.7%) (Table S7) [55]. Among the female population, there were large decreases in GBC incidence in Norway (−11.5%), Italy (−5.7%) and Spain (−5.0%) (Table S7) [55]. Large increases in GBC incidence were observed in the male population in Northern Ireland (13.1%), Italy (5.0%) and France (4.5%) and in the female population in Thailand (8.9%) and France (2.9%) (Table S7) [55].

Very few studies investigated trends in the incidence of AC. Data from France show a sharp decline in the incidence of AC in males (−14.6%) and females (−16.6%) between 2012 and 2019 (Table 2), while data for the US show much smaller decreases between 2001 and 2015 (−0.14% in males and −0.08% in females; Table 2) [12, 37].

### Prevalence

While several articles reported on the incidence of BTC, there were notably fewer studies reporting on prevalence. Exceptions included one US-based study, in which the 10-year ASR of prevalence of BTC was reported for 2015 as 10.8 per 100,000 persons (Table S9, see ESM) [12]. One study conducted in China (2019) recorded the 1-year prevalence of BTC as 2.40 per 100,000 persons (Table S10, see ESM) [41].

The prevalence of CCA has been reported separately for iCCA and eCCA in a small number of individual studies. In the US, the 5-year prevalence (2007–2012) of iCCA and eCCA were estimated as 0.98/100,000 and 2.96/100,000, respectively (Table S9, see ESM) [43]. However, a second US-based study reported a somewhat different pattern, with 10-year prevalence rates in 2015 of 2.5/100,000 and 2.0/100,000 for iCCA and eCCA, respectively (Table S9) [12].

For GBC, 5-year prevalence rates per 100,000 persons are relatively high in Asia (with examples ranging from 2.3 in China to 8.0 in South Korea), followed by Europe (examples ranging from 1.1 in Norway and France to 2.9 in Finland) and the US (1.7) (Table S8, see ESM) [39].

Limited data are available for AC; however, the 10-year prevalence in the US (2005–2015) was estimated as 2.8 per 100,000 persons (Table S9, see ESM) [12].

### Frequency of MDM2 Amplification

Data on the frequency of MDM2 amplification, based on DNA analyses such as DNA sequencing, two-colour in situ hybridisation and fluorescent in situ hybridisation, were identified in 19 individual sources. However, only four provided a definition of MDM2 amplification. A summary of the included publications is presented in Table S2 (see ESM).

**Fig. 3:**
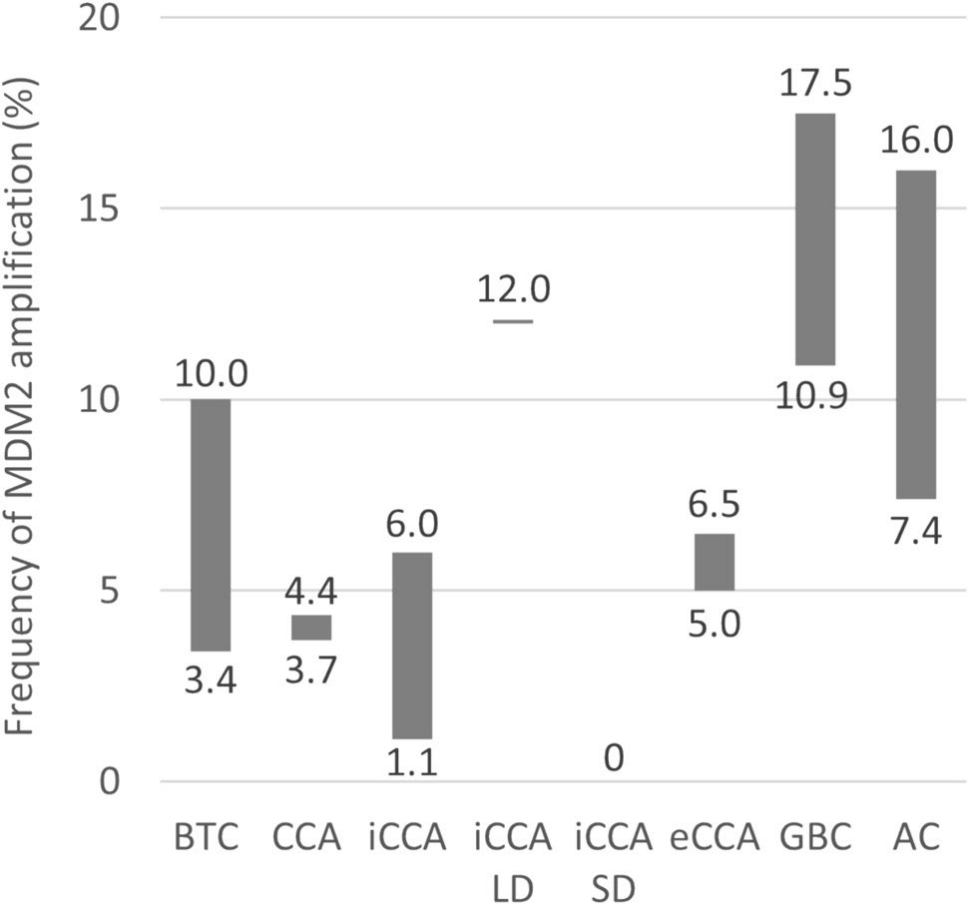
Range of MDM2 amplification frequency in BTC reported across studies [56–59, 62–71]. No MDM2 amplifications were detected in small duct iCCA (iCCA SD). Due to the different methods and definitions used in the individual studies, the comparison of MDM2 amplification frequencies should be made with caution. *AC* ampullary cancer, *BTC* biliary tract cancer, *CCA* cholangiocarcinoma, *eCCA* extrahepatic cholangiocarcinoma, *GBC* gallbladder cancer, *iCCA* intrahepatic cholangiocarcinoma, *LD* large duct, *MDM2* mouse double minute 2 homolog, *SD* small duct

Across the studies identified, the frequency of MDM2 amplification varied considerably for BTC overall and the individual primary sites (Fig. 3). The proportion of tumour samples exhibiting MDM2 amplification ranged from 3.4 to 10.0% for BTC overall [56–61]. Among the primary sites for BTC, GBC had highest proportion of samples with MDM2 amplification (10.9–17.5% of GBC samples contained the alteration; N ranged from 40 to 554) [16, 25, 62, 63]. In comparison, only 3.7% (*N* = 270) to 4.4% (*N* not reported) of CCA samples exhibited MDM2 amplification [64, 65].

Studies investigating iCCA reported large variation in MDM2 amplification frequency ranging from 1.1% to 6.0% (*N* ranged from 92 to 6130) [16, 63, 64, 66, 67]. Interestingly, in one study that reported on iCCA subtypes (small and large duct iCCA), all MDM2 amplifications were identified in the large duct samples and none in the small duct samples [63]. For eCCA, MDM2 amplification frequency in one study ranged from 5% to 6.5% (*N* ranged from 62 to 178) [16, 63, 64, 68].

Across studies for AC, estimates for MDM2 amplification frequency varied greatly, ranging from 7.4 to 16.0% of AC samples (*N* ranged from 45 to 54) [65, 69, 70].

### Biliary Tract Cancer Classifications and Definitions

The majority of studies identified in this review reported the ICD-10/ICD-O-3 topography codes used (Table S1, see ESM). Studies describing the epidemiology of iCCA, eCCA and GBC were generally consistent in their use of ICD site codes. One exception was whether ACs and Klatskin tumours were included in the definition of CCA or reported separately [12, 36, 42, 43]. Additionally, some studies included non-specific ICD-10 codes C24.8 (overlapping lesion of biliary tract) and 24.9 (biliary tract, unspecified) in the definition of cancer sites, making comparisons across studies more difficult [12, 36, 47, 50, 72, 73]. Importantly, however, six studies did not report ICD-10 topography codes, and many studies did not report morphology codes, which allow further delineation of the histologic type of the tumour [74, 75]. Among the studies that did report morphology codes, there was considerable variation in the codes used to define the patient population. The identified variation in the topography and morphology codes used may explain discrepancies in the results published by different independent studies.

## Discussion

### Epidemiology of Biliary Tract Cancer

BTC is a rare and heterogeneous disease for which there is a corresponding paucity of detailed epidemiological studies. Given the poor prognosis of the disease, it is important to understand its prevalence and identify temporal changes in incidence, both for BTC overall and for individual primary sites. In an effort to investigate this, we synthesised evidence from epidemiological studies published between 1 January 2020 and 11 September 2023. Notably, data interpretation and the ability to synthesise findings across studies were hampered by inconsistencies in reporting of ICD and morphology codes, and the populations used for age standardisation (country-specific vs global) [12, 42, 43]. In particular, the inconsistent use of non-specific ICD-10 codes 24.8 (overlapping lesion of biliary tract) and 24.9 (biliary tract unspecified) can lead to inaccurate reporting of incidence and prevalence data. Conflicting reports on the incidence of eCCA in the US include an annualised estimate of 0.74 per 100,000 in 2000–2017 in one study, while a second study provides an annualised estimate for 2001–2017 of 2.46 per 100,000 (Table 1) [42, 43]. These studies appear to apply different levels of stringency with respect to the definitions of eCCA used (Table S2, see ESM).

Collectively, data support wide geographic variation in the incidence of BTC anatomic subtypes. Incidence rates of both CCA and GBC were highest in South and Central Asia compared with other regions of the world [35]. Data on the incidence of AC were sparse and inconclusive, with comparatively low incidence rates observed across all regions of interest [40].

While further investigation in case-controlled observational studies is needed, elevated incidence of CCA and GBC in parts of Asia is consistent with higher exposure to certain risk factors including parasitic fluke infections, and a higher incidence of hepatolithiasis (bile duct stones) and gallstones [76, 77]. The correlation between high incidence of gallstones and GBC is already well documented in studies from different countries in Asia and South America, notably Chile, where rates of GBC among females are among the highest in the world [40, 78].

When looking at differences in BTC incidence according to sex, there was a global trend toward higher GBC incidence in females compared with males, possibly related to the higher prevalence of gallstones in females [78–80]. Interestingly, this trend differed in some Asian countries, where rates of GBC were higher in males compared with females (South Korea and Thailand, 2020), or were equivalent between sexes (Japan, 2016–2017) [39, 46].

### MDM2 Amplification in Biliary Tract Cancers

Genomic alterations, including those which are therapeutically actionable, vary in frequency among anatomic subtypes of BTC [13–15]. Consequently, the coupling of accurate clinical diagnostics to molecular profiling becomes increasingly important [16]. The value of molecular profiling according to primary site is already being realised through the exploration of targeted therapies for the treatment of specific molecular subtypes of BTC, including aberrations in *FGFR2, IDH1, BRAF V600E*, *HER2* and *RET* [3, 13, 81]. MDM2 represents a novel drug target for the treatment of BTC, with early phase trials already investigating MDM2 inhibitors brigimadlin and milademetan in this indication [28, 29]. This literature review found that the proportion of tumours exhibiting MDM2 amplification ranged from 3.4 to 10.0% for BTC overall [56–61]. Among primary sites, high rates of MDM2 amplification were consistently observed in GBC samples, while lower rates of MDM2 amplification were seen in CCA samples; the frequency of amplification in AC samples varied [62, 63, 69, 70]. However, comparing these results should be done with caution, as the methods used to assess genetic aberrations varied between studies, there is significant risk for site-specific selection bias, and only few publications provided the definitions of MDM2 amplification used.

### Limitations and Strengths of this Review

This review provides a synthesis of evidence on the epidemiology of BTC and the frequency of MDM2 amplification in BTC tumours. Few literature reviews have been published on this topic and therefore we believe that this research helps to form a better understanding of the incidence and prevalence of BTC and identify current evidence gaps.

As this was a targeted literature review, the objective was to identify publications most relevant to the research questions, rather than to carry out a systematic and comprehensive review of all published literature. As such, the retrieved evidence was not formally qualitatively appraised. A review of the screening and data extraction process was performed by a second researcher to mitigate study selection bias.

### Evidence Gaps and Implications for Future Research

This literature review identified very limited data for AC and for the two subtypes of eCCA (distal CCA [dCCA] and perihilar CCA [pCCA]). Differences in the molecular biology of these tumour subtypes may impact their susceptibility to therapy, and therefore an effort should be made to increase our knowledge of subtype epidemiology [16].

Prevalence data are particularly limited compared with data for incidence, with no prevalence data for CCA outside of the US [43]. The broadest data set for prevalence was obtained from IARC, which highlighted discrete regions of relatively high 5-year prevalence of GBC in China, Japan and South Korea, the UK, Norway and Finland (Table S4, see ESM) [39].

With respect to MDM2 amplification, studies for BTC often did not provide the definition used for amplification, with few studies providing details regarding the copy number. The combination of differences in methods used to determine amplification status and the absence of a common definition for MDM2 amplification limits our ability to compare studies. The molecular heterogeneity seen by primary tumour site should be explored further across large registry studies that include comprehensive molecular profiling as a clear opportunity for future research.

## Conclusions

As the number of BTC cases continues to rise in some parts of the globe, an understanding of disease risk factors, aetiology and potential therapeutic avenues becomes increasingly important. Although this review did not explore literature on mortality, evidence supporting variation in the overall survival rates of patients with BTC according to anatomic subtype further emphasises the need for accurate epidemiologic data [16]. Going forward, consistent reporting of ICD codes will be important for cross-study comparisons of data for BTC.

The implementation of the 11th revision of the ICD coding system, which has been in effect since January 2022, is being facilitated by the World Health Organization (WHO) and gradually being adopted by 132 WHO Member States, with 50 countries currently piloting the implementation and 14 countries now using ICD-11 coding for data collection [82]. This updated coding system aims to provide a more precise and rigorous approach to coding BTC, taking into account the diverse topography and morphology of tumours [83]. Standardising the clinicopathologic assessment and reporting of BTC is important, as the clinical diversity of anatomical BTC subtypes influences patient management [3]. This is already reflected in clinical guidelines which support the use of sensitive cross-sectional imaging and routine endoscopic evaluation to localise the primary cancer site [3, 84].

## Supplementary Information

Below is the link to the electronic supplementary material.

Supplementary file1 (PDF 268 KB)

## Declarations

### Availability of Data and Materials

The majority of data described in this article was sourced from published works as cited throughout. In addition, the following databases were used and are cited where appropriate: International Agency for Research on Cancer (IARC); Globocan 2022 (version 1.1), available here: https://gco.iarc.who.int. National Cancer Institute. Surveillance, Epidemiology, and End Results Program (SEER). SEER*Explorer, available here: https://seer.cancer.gov/statistics-network/explorer/. My Cancer Genome^®^. Vanderbilt-Ingram Cancer Center, available here: https://www.mycancergenome.org/.

## References

[1] Valle JW, Kelley RK, Nervi B, Oh DY, Zhu AX. Biliary tract cancer. Lancet. 2021;397(10272):428–44. 10.1016/S0140-6736(21)00153-7. 33516341 10.1016/S0140-6736(21)00153-7

[2] World Health Organization (WHO), ICD-10 Version: 2019. https://icd.who.int/browse10/2019/en. Accessed 4th April. 2023.

[3] Vogel A, Bridgewater J, Edeline J, Kelley RK, Klumpen HJ, Malka D, et al. Biliary tract cancer: ESMO Clinical Practice Guideline for diagnosis, treatment and follow-up. Ann Oncol. 2023;34(2):127–40. 10.1016/j.annonc.2022.10.506. 36372281 10.1016/j.annonc.2022.10.506

[4] Clements O, Eliahoo J, Kim JU, Taylor-Robinson SD, Khan SA. Risk factors for intrahepatic and extrahepatic cholangiocarcinoma: A systematic review and meta-analysis. J Hepatol. 2020;72(1):95–103. 10.1016/j.jhep.2019.09.007. 31536748 10.1016/j.jhep.2019.09.007

[5] Kamsa-ard S, Kamsa-ard S, Luvira V, Suwanrungruang K, Vatanasapt P, Wiangnon S. Risk factors for cholangiocarcinoma in Thailand: a systematic review and meta-analysis. Asian Pac J Cancer Prev. 2018;19(3):605–14. 10.22034/APJCP.2018.19.3.605. 29579789 10.22034/APJCP.2018.19.3.605 PMC5980830

[6] Hsing AW, Gao YT, Han TQ, Rashid A, Sakoda LC, Wang BS, et al. Gallstones and the risk of biliary tract cancer: a population-based study in China. Br J Cancer. 2007;97(11):1577–82. 10.1038/sj.bjc.6604047. 18000509 10.1038/sj.bjc.6604047 PMC2360257

[7] Nagaraja V, Eslick GD. Systematic review with meta-analysis: the relationship between chronic Salmonella typhi carrier status and gall-bladder cancer. Aliment Pharmacol Ther. 2014;39(8):745–50. 10.1111/apt.12655. 24612190 10.1111/apt.12655

[8] He XD, Wu Q, Liu W, Hong T, Li JJ, Miao RY, Zhao HT. Association of metabolic syndromes and risk factors with ampullary tumors development: a case-control study in China. World J Gastroenterol. 2014;20(28):9541–8. 10.3748/wjg.v20.i28.9541. 25071350 10.3748/wjg.v20.i28.9541 PMC4110587

[9] Villard C, Friis-Liby I, Rorsman F, Said K, Warnqvist A, Cornillet M, et al. Prospective surveillance for cholangiocarcinoma in unselected individuals with primary sclerosing cholangitis. J Hepatol. 2023;78(3):604–13. 10.1016/j.jhep.2022.11.011. 36410555 10.1016/j.jhep.2022.11.011

[10] Brindley PJ, Bachini M, Ilyas SI, Khan SA, Loukas A, Sirica AE, et al. Cholangiocarcinoma. Nat Rev Dis Primers. 2021;7(1):65. 10.1038/s41572-021-00300-2. 34504109 10.1038/s41572-021-00300-2 PMC9246479

[11] Valle JW, Lamarca A, Goyal L, Barriuso J, Zhu AX. New horizons for precision medicine in biliary tract cancers. Cancer Discov. 2017;7(9):943–62. 10.1158/2159-8290.CD-17-0245. 28818953 10.1158/2159-8290.CD-17-0245 PMC5586506

[12] Koshiol J, Yu B, Kabadi SM, Baria K, Shroff RT. Epidemiologic patterns of biliary tract cancer in the United States: 2001–2015. BMC Cancer. 2022;22(1):1178. 10.1186/s12885-022-10286-z. 36384474 10.1186/s12885-022-10286-z PMC9670575

[13] Scott AJ, Sharman R, Shroff RT. Precision medicine in biliary tract cancer. J Clin Oncol. 2022;40(24):2716–34. 10.1200/jco.21.02576. 35839428 10.1200/JCO.21.02576

[14] Weinberg BA, Xiu J, Lindberg MR, Shields AF, Hwang JJ, Poorman K, et al. Molecular profiling of biliary cancers reveals distinct molecular alterations and potential therapeutic targets. J Gastrointest Oncol. 2019;10(4):652–62. 10.21037/jgo.2018.08.18. 31392046 10.21037/jgo.2018.08.18 PMC6657312

[15] Jusakul A, Cutcutache I, Yong CH, Lim JQ, Huang MN, Padmanabhan N, et al. Whole-genome and epigenomic landscapes of etiologically distinct subtypes of cholangiocarcinoma. Cancer Discov. 2017;7(10):1116–35. 10.1158/2159-8290.Cd-17-0368. 28667006 10.1158/2159-8290.CD-17-0368 PMC5628134

[16] Lin ES, Mehlhaff EG, Bergstrom CP, Lesnik DM, LoConte NK, Lubner SJ, et al. Actionable molecular alterations in veterans with advanced cholangiocarcinoma. J Clin Oncol. 2024;42:548.

[17] Giraldo NA, Drill E, Satravada BA, Dika IE, Brannon AR, Dermawan J, et al. Comprehensive molecular characterization of gallbladder carcinoma and potential targets for intervention. Clin Cancer Res. 2022;28(24):5359–67. 10.1158/1078-0432.Ccr-22-1954. 36228155 10.1158/1078-0432.CCR-22-1954 PMC9772093

[18] Mody K, Jain P, El-Refai SM, Azad NS, Zabransky DJ, Baretti M, et al. Clinical, genomic, and transcriptomic data profiling of biliary tract cancer reveals subtype-specific immune signatures. JCO Precis Oncol. 2022;6: e2100510. 10.1200/po.21.00510. 35675577 10.1200/PO.21.00510 PMC9200391

[19] Lee H, Ross JS. The potential role of comprehensive genomic profiling to guide targeted therapy for patients with biliary cancer. Therap Adv Gastroenterol. 2017;10(6):507–20. 10.1177/1756283X17698090. 28567120 10.1177/1756283X17698090 PMC5424872

[20] Yoo KH, Kim NK, Kwon WI, Lee C, Kim SY, Jang J, et al. Genomic alterations in biliary tract cancer using targeted sequencing. Transl Oncol. 2016;9(3):173–8. 10.1016/j.tranon.2016.01.007. 27267833 10.1016/j.tranon.2016.01.007 PMC4856857

[21] Jain A, Javle M. Molecular profiling of biliary tract cancer: a target rich disease. J Gastrointest Oncol. 2016;7(5):797–803. 10.21037/jgo.2016.09.01. 27747093 10.21037/jgo.2016.09.01 PMC5056250

[22] Oliner JD, Saiki AY, Caenepeel S. The role of MDM2 amplification and overexpression in tumorigenesis. Cold Spring Harb Perspect Med. 2016. 10.1101/cshperspect.a026336. 27194168 10.1101/cshperspect.a026336 PMC4888815

[23] Zhu H, Gao H, Ji Y, Zhou Q, Du Z, Tian L, et al. Targeting p53-MDM2 interaction by small-molecule inhibitors: learning from MDM2 inhibitors in clinical trials. J Hematol Oncol. 2022;15(1):91. 10.1186/s13045-022-01314-3. 35831864 10.1186/s13045-022-01314-3 PMC9277894

[24] Zhao Y, Yu H, Hu W. The regulation of MDM2 oncogene and its impact on human cancers. Acta Biochim Biophys Sin (Shanghai). 2014;46(3):180–9. 10.1093/abbs/gmt147. 24389645 10.1093/abbs/gmt147 PMC3932834

[25] Kato S, Ross JS, Gay L, Dayyani F, Roszik J, Subbiah V, Kurzrock R. Analysis of MDM2 amplification: next-generation sequencing of patients with diverse malignancies. JCO Precis Oncol. 2018. 10.1200/PO.17.00235. 30148248 10.1200/PO.17.00235 PMC6106866

[26] Momand J, Jung D, Wilczynski S, Niland J. The MDM2 gene amplification database. Nucleic Acids Res. 1998;26(15):3453–9. 10.1093/nar/26.15.3453. 9671804 10.1093/nar/26.15.3453 PMC147746

[27] Dembla V, Somaiah N, Barata P, Hess K, Fu S, Janku F, et al. Prevalence of MDM2 amplification and coalterations in 523 advanced cancer patients in the MD Anderson phase 1 clinic. Oncotarget. 2018;9(69):33232–43. 10.18632/oncotarget.26075. 30237864 10.18632/oncotarget.26075 PMC6145698

[28] Yamamoto N, Tolcher AW, Hafez N, Lugowska I, Ramlau R, Gounder MM, et al. Efficacy and safety of the MDM2-p53 antagonist BI 907828 in patients with advanced biliary tract cancer: data from two phase Ia/Ib dose-escalation/expansion trials. J Clin Oncol. 2023;41(4 Supplement):543. 10.1200/jco.2023.41.4_suppl.543.

[29] Dumbrava EE, Chen CT, Cote GM, Hanna GJ, Stinchcombe TE, Sumrall B, et al. Abstract B034: A phase 2 basket study of the oral MDM2 inhibitor milademetan for MDM2-amplified advanced solid tumors (MANTRA-2). Mol Cancer Therapeutics. 2023;22(12_Supplement):B034–B034. 10.1158/1535-7163.Targ-23-b034.

[30] ClinicalTrials.gov, NCT03975387. Study of ASTX295 in patients with solid tumors with wild-type p53. https://clinicaltrials.gov/study/NCT03975387?intr=ASTX-295&rank=1#study-overview. Accessed 7th Feb 2024.

[31] ClinicalTrials.gov, NCT05775406. Safety and clinical activity of KT-253 in adult patients with high grade myeloid malignancies, acute lymphocytic Leukemia, Lymphoma, Solid Tumors. https://clinicaltrials.gov/study/NCT05775406?intr=KT-253&rank=1. Accessed 7th Feb 2024.

[32] Xie W, Yang T, Zuo J, Ma Z, Yu W, Hu Z, Song Z. Chinese and global burdens of gastrointestinal cancers from 1990 to 2019. Front Public Health. 2022;10: 941284. 10.3389/fpubh.2022.941284. 35910886 10.3389/fpubh.2022.941284 PMC9326121

[33] Florio AA, Ferlay J, Znaor A, Ruggieri D, Alvarez CS, Laversanne M, et al. Global trends in intrahepatic and extrahepatic cholangiocarcinoma incidence from 1993 to 2012. Cancer. 2020;126(11):2666–78. 10.1002/cncr.32803. 32129902 10.1002/cncr.32803 PMC7323858

[34] Rumgay H, Ferlay J, de Martel C, Georges D, Ibrahim AS, Zheng R, et al. Global, regional and national burden of primary liver cancer by subtype. Eur J Cancer. 2022;161:108–18. 10.1016/j.ejca.2021.11.023. 34942552 10.1016/j.ejca.2021.11.023

[35] Miranda-Filho A, Pineros M, Ferreccio C, Adsay V, Soerjomataram I, Bray F, Koshiol J. Gallbladder and extrahepatic bile duct cancers in the Americas: incidence and mortality patterns and trends. Int J Cancer. 2020;147(4):978–89. 10.1002/ijc.32863. 31922259 10.1002/ijc.32863 PMC8629410

[36] Rahman R, Ludvigsson JF, von Seth E, Lagergren J, Bergquist A, Radkiewicz C. Age trends in biliary tract cancer incidence by anatomical subtype: a Swedish cohort study. Eur J Cancer. 2022;175:291–8. 10.1016/j.ejca.2022.08.032. 36174301 10.1016/j.ejca.2022.08.032

[37] Ghiringhelli F, Jooste V, Manfredi S, Hennequin A, Lepage C, Bouvier AM. Biliary tract cancers have distinct epidemiological patterns and clinical characteristics according to tumour site. HPB (Oxford). 2023. 10.1016/j.hpb.2023.02.016. 36958986 10.1016/j.hpb.2023.02.016

[38] Kang MJ, Yun EH, Jung KW, Park SJ. Incidence, mortality and survival of gallbladder, extrahepatic bile duct, and pancreatic cancer using Korea central cancer registry database: 1999–2019. Ann Hepatobiliary Pancreat Surg. 2022;26(3):220–8. 10.14701/ahbps.22-041. 35909086 10.14701/ahbps.22-041 PMC9428428

[39] Ferlay J, Ervik M, Lam F, Laversanne M, Colombet M, Mery L et al. Global Cancer Observatory: Cancer Today. Lyon, France: International Agency for Research on Cancer. https://gco.iarc.who.int/today. Accessed 30 Mar 2023.

[40] Baria K, De Toni E, Yu B, Jiang Z, Kabadi S, Malvezz M. Worldwide incidence and mortality of biliary tract cancer. Gastro Hep Adv. 2022;1(4):618–26. 39132071 10.1016/j.gastha.2022.04.007 PMC11307584

[41] Chen S, Han K, Song Y, Liu S, Li X, Wang S, et al. Current status, trends, and predictions in the burden of gallbladder and biliary tract cancer in China from 1990 to 2019. Chin Med J (Engl). 2022;135(14):1697–706. 10.1097/CM9.0000000000002258. 35984211 10.1097/CM9.0000000000002258 PMC9509182

[42] Tella SH, Wieczorek M, Hodge D, Mahipal A. A glimpse into the future of cholangiocarcinoma: predicting the future incidence based on the current epidemiological data. J Clin Oncol. 2023;41(4_suppl):616–616. 10.1200/JCO.2023.41.4_suppl.616.

[43] Javle M, Lee S, Azad NS, Borad MJ, Kate Kelley R, Sivaraman S, et al. Temporal changes in cholangiocarcinoma incidence and mortality in the United States from 2001 to 2017. Oncologist. 2022;27(10):874–83. 10.1093/oncolo/oyac150. 35972334 10.1093/oncolo/oyac150 PMC9526482

[44] Hong SY, Kang MJ, Kim T, Jung KW, Kim BW. Incidence, mortality, and survival of liver cancer using Korea central cancer registry database: 1999–2019. Ann Hepatobiliary Pancreat Surg. 2022;26(3):211–9. 10.14701/ahbps.22-044. 35934831 10.14701/ahbps.22-044 PMC9428436

[45] SEER*Explorer. An interactive website for SEER cancer statistics. Surveillance Research Program, National Cancer Institute. https://seer.cancer.gov/statistics-network/explorer/. Accessed Jan 2024.

[46] Makiuchi T, Sobue T. Descriptive epidemiology of biliary tract cancer incidence and geographic variation in Japan. Eur J Cancer Prev. 2023;32(1):2–9. 10.1097/CEJ.0000000000000758. 35485392 10.1097/CEJ.0000000000000758

[47] Barner-Rasmussen N, Pukkala E, Hadkhale K, Farkkila M. Risk factors, epidemiology and prognosis of cholangiocarcinoma in Finland. United Eur Gastroenterol J. 2021;9(10):1128–35. 10.1002/ueg2.12154. 10.1002/ueg2.12154 PMC8672081 34533900

[48] Zhu MX, Li Y. The correlations between socioeconomic status and intrahepatic cholangiocarcinoma in the United States: a population-based study. Transl Cancer Res. 2020;9(8):4931–42. 10.21037/tcr-20-2506. 35117855 10.21037/tcr-20-2506 PMC8798916

[49] Mancini S, Bucchi L, Zamagni F, Guzzinati S, Dal Maso L, Rugge M, et al. Trends in liver cancer incidence and survival in Italy by histologic type, 2003–2017. Cancers (Basel). 2022. 10.3390/cancers14246162. 36551647 10.3390/cancers14246162 PMC9777051

[50] Kamsa-Ard S, Santong C, Kamsa-Ard S, Luvira V, Luvira V, Suwanrungruang K, Bhudhisawasdi V. Decreasing trends in cholangiocarcinoma incidence and relative survival in Khon Kaen, Thailand: an updated, inclusive, population-based cancer registry analysis for 1989–2018. PLoS One. 2021;16(2): e0246490. 10.1371/journal.pone.0246490. 33592053 10.1371/journal.pone.0246490 PMC7886206

[51] Raza SA, da Costa WL, Thrift AP. Increasing incidence of gallbladder cancer among non-hispanic blacks in the United States: a birth cohort phenomenon. Cancer Epidemiol Biomarkers Prev. 2022;31(7):1410–7. 10.1158/1055-9965.EPI-21-1452. 35437571 10.1158/1055-9965.EPI-21-1452

[52] An L, Zheng R, Zhang S, Chen R, Wang S, Sun K, et al. Hepatocellular carcinoma and intrahepatic cholangiocarcinoma incidence between 2006 and 2015 in China: estimates based on data from 188 population-based cancer registries. Hepatobiliary Surg Nutr. 2023;12(1):45–55. 10.21037/hbsn-21-75. 36860251 10.21037/hbsn-21-75 PMC9944524

[53] Ali H, Tedder B, Waqar SH, Mohamed R, Cate EL, Ali E. Changing incidence and survival of intrahepatic cholangiocarcinoma based on surveillance, epidemiology, and end results database (2000–2017). Ann Hepatobiliary Pancreat Surg. 2022;26(3):235–43. 10.14701/ahbps.21-173. 35811455 10.14701/ahbps.21-173 PMC9428430

[54] Xing H, Tan B, Yang C, Zhang M. Incidence trend and competing risk analysis of patients with intrahepatic cholangiocarcinoma: a population-based study. Front Med (Lausanne). 2022;9: 846276. 10.3389/fmed.2022.846276. 35433765 10.3389/fmed.2022.846276 PMC9005886

[55] Ervik M, Lam F, Laversanne M, Ferlay J and Bray F. Global cancer observatory: cancer over time. Lyon, France: International Agency for Research on Cancer. https://gco.iarc.fr/overtime. Accessed 30th Mar 2023.

[56] Battaglin F, Xiu J, Baca Y, Shields AF, Goldberg RM, Puccini A, et al. Comprehensive profiling of MDM2 amplified gastrointestinal (GI) cancers. Ann Oncol. 2020;31(Supplement 4):S1100. 10.1016/j.annonc.2020.08.1344.

[57] Cassier P, De La Fouchardiere C, Guibert P, Pissaloux D, Pacaux C, Terret C, et al. Actionable molecular alterations in advanced biliary tract carcinomas: preliminary data from the ProfiLER program (NCT01774409). Ann Oncol. 2017;28(Supplement 5): v247. 10.1093/annonc/mdx369.109.

[58] Lin J, Yang X, Cao Y, Li G, Zhao S, Shi J, et al. Genomics and translational precision oncology for 803 patients with biliary tract cancer. J Clin Oncol Conf. 2020. 10.1200/JCO.2020.38.15_suppl.4589.

[59] Bouattour M, Juan W, Valle JW, Vogel A, Kim JW, Kitano M et al. Characterization of long-term survivors in the TOPAZ-1 study of durvalumab or placebo plus gemcitabine and cisplatin in advanced biliary tract cancer. in ASCO Gastrointestinal Cancers Symposium. 2023. San Francisco, CA.

[60] Rimini M, Rizzato M, Rimassa L, Niger M, Fornaro L, Antonuzzo L, et al. P-343 The impact of genomic alterations on response rate and survival outcomes in advanced BTC patients who receive cisplatin/gemcitabine plus durvalumab in clinical practice. Ann Oncol. 2023;34:S135.

[61] Kumar-Sinha C, Vats P, Tran N, Robinson DR, Gunchick V, Wu YM, et al. Genomics driven precision oncology in advanced biliary tract cancer improves survival. Neoplasia. 2023;42: 100910. 10.1016/j.neo.2023.100910. 37267699 10.1016/j.neo.2023.100910 PMC10245336

[62] D’Afonseca V, Arencibia AD, Echeverria-Vega A, Cerpa L, Cayun JP, Varela NM, et al. Identification of altered genes in gallbladder cancer as potential driver mutations for diagnostic and prognostic purposes: a computational approach. Cancer Inform [Electronic Resource]. 2020;19:1176935120922154. 10.1177/1176935120922154. 32546937 10.1177/1176935120922154 PMC7249562

[63] Kim SJ, Akita M, Sung YN, Fujikura K, Lee JH, Hwang S, et al. MDM2 amplification in intrahepatic cholangiocarcinomas: its relationship with large-duct type morphology and uncommon KRAS mutations. Am J Surg Pathol. 2018;42(4):512–21. 10.1097/PAS.0000000000001006. 29309301 10.1097/PAS.0000000000001006

[64] Zheng Y, Qin Y, Gong W, Li H, Li B, Wang Y, et al. Specific genomic alterations and prognostic analysis of perihilar cholangiocarcinoma and distal cholangiocarcinoma. J Gastrointest Oncol. 2021;12(6):2631–42. 10.21037/jgo-21-776. 35070393 10.21037/jgo-21-776 PMC8748027

[65] MyCancerGenome. Biomarkers- MDM2 Amplification. https://www.mycancergenome.org/content/alteration/mdm2-amplification/. Accessed 30 Mar 2023.

[66] Kendre G, Murugesan K, Brummer T, Segatto O, Saborowski A, Vogel A. Charting co-mutation patterns associated with actionable drivers in intrahepatic cholangiocarcinoma. J Hepatol. 2023;78(3):614–26. 10.1016/j.jhep.2022.11.030. 36528236 10.1016/j.jhep.2022.11.030

[67] Pu X, Zhu L, Li F, Zheng J, Wu H, Fu Y, et al. Target molecular treatment markers in Intrahepatic Cholangiocarcinoma based on Chinese population. Pathol Res Pract. 2020;216(9): 153116. 10.1016/j.prp.2020.153116. 32825971 10.1016/j.prp.2020.153116

[68] Lee H, Wang K, Johnson A, Jones DM, Ali SM, Elvin JA, et al. Comprehensive genomic profiling of extrahepatic cholangiocarcinoma reveals a long tail of therapeutic targets. J Clin Pathol. 2016;69(5):403–8. 10.1136/jclinpath-2015-203394. 26500333 10.1136/jclinpath-2015-203394

[69] Wong W, Lowery MA, Berger MF, Kemel Y, Taylor B, Zehir A, et al. Ampullary cancer: evaluation of somatic and germline genetic alterations and association with clinical outcomes. Cancer. 2019;125(9):1441–8. 10.1002/cncr.31951. 30620386 10.1002/cncr.31951 PMC6467723

[70] Harthimmer MR, Stolborg U, Pfeiffer P, Mortensen MB, Fristrup C, Detlefsen S. Mutational profiling and immunohistochemical analysis of a surgical series of ampullary carcinomas. J Clin Pathol. 2019;72(11):762–70. 10.1136/jclinpath-2019-205912. 31256008 10.1136/jclinpath-2019-205912

[71] Feng F, Wu X, Shi X, Gao Q, Wu Y, Yu Y, et al. Comprehensive analysis of genomic alterations of Chinese hilar cholangiocarcinoma patients. Int J Clin Oncol. 2021;26(4):717–27. 10.1007/s10147-020-01846-z. 33387086 10.1007/s10147-020-01846-z

[72] Cao P, Rozek LS, Pongnikorn D, Sriplung H, Meza R. Comparison of cholangiocarcinoma and hepatocellular carcinoma incidence trends from 1993 to 2012 in Lampang, Thailand. Int J Environ Res Public Health. 2022. 10.3390/ijerph19159551. 35954902 10.3390/ijerph19159551 PMC9368745

[73] Velasco AG, Quintana M, Guinart MP, Carbajal W, Sanchez RG, Anna V, et al. P-216 Incidence and trends of biliary tract cancer in Girona: a population-based study from the Girona Cancer Registry (1994–2016). Ann Oncol. 2020;31:S160–1. 10.1016/j.annonc.2020.04.298.

[74] World Health Organization (WHO). International classification of diseases for oncology, 3rd edition (ICD-O-3). https://www.who.int/standards/classifications/other-classifications/international-classification-of-diseases-for-oncology. Accessed 6th Apr 2023.

[75] National Cancer Institute. Surveillance, epidemiology, and end results program. ICD-O-3 Coding Materials. https://seer.cancer.gov/icd-o-3/. Accessed 4th Apr 2023.

[76] Sithithaworn P, Yongvanit P, Duenngai K, Kiatsopit N, Pairojkul C. Roles of liver fluke infection as risk factor for cholangiocarcinoma. J Hepatobiliary Pancreat Sci. 2014;21(5):301–8. 10.1002/jhbp.62. 24408775 10.1002/jhbp.62

[77] Kim HJ, Kim JS, Joo MK, Lee BJ, Kim JH, Yeon JE, et al. Hepatolithiasis and intrahepatic cholangiocarcinoma: a review. World J Gastroenterol. 2015;21(48):13418–31. 10.3748/wjg.v21.i48.13418. 26730152 10.3748/wjg.v21.i48.13418 PMC4690170

[78] Lazcano-Ponce EC, Miquel JF, Muñoz N, Herrero R, Ferrecio C, Wistuba II, et al. Epidemiology and molecular pathology of gallbladder cancer. Cancer J Clin. 2001;51(6):349–64. 10.3322/canjclin.51.6.349. 10.3322/canjclin.51.6.349 11760569

[79] Alkhayyat M, Abou Saleh M, Qapaja T, Abureesh M, Almomani A, Mansoor E, Chahal P. Epidemiology of gallbladder cancer in the Unites States: a population-based study. Chin Clin Oncol. 2021;10(3):25. 10.21037/cco-20-230. 33615799 10.21037/cco-20-230

[80] Pérez-Moreno P, Riquelme I, García P, Brebi P, Roa JC. Environmental and lifestyle risk factors in the carcinogenesis of gallbladder cancer. J Pers Med. 2022. 10.3390/jpm12020234. 35207722 10.3390/jpm12020234 PMC8877116

[81] National Comprehensive Cancer Network. NCCN Clinical Practice Guidelines in Oncology - Biliary Tract Cancers Version 3. 2023.

[82] World Health Organization (WHO). WHO Advances Implementation and Integration of ICD-11 and Related Medical Classifications and Terminologies. https://www.who.int/standards/classifications/classification-of-diseases. Accessed 11th Jul 2024.

[83] Cai S, Sivakumar S. The 11th revision of the international statistical classification of disease and related health problems and cholangiocarcinoma. Hepatobiliary Surg Nutr. 2022;11(2):276–9. 10.21037/hbsn-22-69. 35464287 10.21037/hbsn-22-69 PMC9023819

[84] Benson AB, D’Angelica MI, Abbott DE, Anaya DA, Anders R, Are C, et al. Hepatobiliary cancers, version 2.2021, NCCN clinical practice guidelines in oncology. J Natl Compr Canc Netw. 2021;19(5):541–65. 10.6004/jnccn.2021.0022. 34030131 10.6004/jnccn.2021.0022

