# Supplementary material for: The Epidemiology of Biliary Tract Cancer and Associated Prevalence of MDM2 Amplification: A Targeted Literature Review
Source: Target Oncol. 2024 Sep 20;19(6):833–44. doi: 10.1007/s11523-024-01086-5 (PMC11557622; doi:10.1007/s11523-024-01086-5)
Supplement: Supplementary file 1 — Supplementary file1 (PDF 268 KB) [file 11523_2024_1086_MOESM1_ESM.pdf]

Supplemental Information

Article title: The Epidemiology of Biliary Tract Cancer and Associated Prevalence of MDM2 Amplification: A Targeted Literature Review

Journal: Targeted Oncology

Authors: Jeremy David Kratz, Alyssa Barchet Klein, Courtney Beth Gray, Angela Märten, Hanna-Liisa Vilu, Jennifer Francesca Knight, Alexandra Kumichel, Makoto Ueno

Corresponding author: Jeremy David Kratz. Department of Medicine, University of Wisconsin, WI, United States. E-mail: jdkratz@medicine.wisc.edu

Table S1: Characteristics of studies included in the review of BTC epidemiology

Study	Study type/ data source	Study location	ICD-10/ICD-O-3 code(s)	Morphology codes	BTC-specific morphology codes
Ali 2022 [1]	SEER-18	US	Intrahepatic bile duct: C22.1	NR	NR
Alkhayyat 2021 [2]	Explorys' EHR data	US	GBC, ICD codes not reported	NR	NR
An 2023 [3]	National Central Cancer Registry, China	China	iCCA: 22.0, 22.1	**C22.0** (8140, 8160, 8161, 8162, 8480, 8481, 8500)<br>**C22.1** (8000, 8010, 8020, 8140, 8160, 8161, 8162, 8480, 8481, 8500)	NR
Baria 2022 [4]	IARC CI5-XI and US National Program of Cancer Registries	Global	iCCA, eCCA, GBC, AC, ICD codes not reported	NR	NR
Barner-Rasmussen 2021 [5]	Finnish Cancer Registry	Finland	CCA: C22.1, C24.0, C24.8, C24.9<br>iCCA; C22.1<br>eCCA: C24.0	8000, 8010, 8020-8021, 8050, 8070, 8140, 8148, 8160-8162, 8180, 8260, 8440, 8460 and 8560	8160/0: Bile duct adenoma<br>8160/3: CCA<br>8161/0: Bile duct cystadenoma (iCCA)<br>8161/3: Bile duct cystadenocarcinoma<br>8162/3: Perihilar CCA[a], Klatskin tumour[a]<br>8180/3: Combined hepatocellular carcinoma and CCA
Cao 2022 [6]	Lampang cancer registry	Thailand	CCA: C22.1, C24.0, C24.8 or C24.9	NR	NR
Chen 2022 [7]	Global Burden of Disease Study 2019	China	BTC + GBC, ICD codes not reported	NR	NR
Florio 2020 [8]	CI5*plus* cancer registry	Global	iCCA: C22.0<br>eCCA: C22.0 or C24.0	**eCCA:**<br>**C22.0** (8162)<br>**C24.0** (8050, 8140-8141, 8160-8161, 8260, 8440, 8480-8500, 8570-8572, 8162)<br>**iCCA:**<br>**C22.0** (8050, 8140-8141, 8160-8161, 8260, 8440, 8480, 8480-8500, 8570-8572)	**C24.0**<br>8160/0: Bile duct adenoma<br>8160/3: CCA<br>8161/3: Bile duct cystadenocarcinoma<br>8162/3: Perihilar CCA[a], Klatskin tumour[a]<br>**C22.0** N/A
Ghiringhelli 2023 [9]	Digestive Cancer Registry of Burgundy	France	AC: C24.1<br>iCCA: C22.1<br>eCCA: C24.0 + Klatskin tumours[a]<br>GBC: C23.0	8000, 8010, 8020, 8050, 8140, 8160, 8260, 8440, 8480, 8490, and 8560; 8162/3	8160/0: Bile duct adenoma<br>8160/3: CCA<br>8162/3: Perihilar CCA[a], Klatskin tumour[a]

Study	Study type/ data source	Study location	ICD-10/ICD-O-3 code(s)	Morphology codes	BTC-specific morphology codes
GLOBOCAN [10]		Global	GBC: 23.0	NR	NR
Hong 2022 [11]	KCCR based on the Korea National Cancer Incidence Database	Korea	iCCA: C22.1	NR	NR
Javle 2022 [12]	SEER-18	US	iCCA: C22.0, C22.1<br>eCCA: C24.1, C22.0, C22.1 or C24.0, C24.9	**eCCA:**<br>**C24.1** with 8000, 8010, 8020, 8140, 8160, 8161, 8260, 8480, 8481, 8490, 8500<br>**C22.0, C22.1, or C24.0** with 8162<br>**iCCA:**<br>**C22.0** (8140, 8160, 8161, 8480, 8481, 8500)<br>**C22.1** (8000, 8010, 8020, 8140, 8160, 8161, 8260, 8480, 8481, 8490, or 8500)	8160/0: Bile duct adenoma<br>8160/3: CCA<br>8161/0: Bile duct cystadenoma<br>8161/3: Bile duct cystadenocarcinoma<br>8162/3: Perihilar CCA[a], Klatskin tumour[a] (eCCA)
Kamsa-Ard 2021 [13]	KKCR, Northeast Thailand	Thailand	CCA: C22.1, C24.0, C24.8, C24.9 *(C24.1 excluded)*	NR	NR
Kang 2022 [14]	KCCR based on the Korea National Cancer Incidence Database	Korea	eCCA: C24.0<br>GBC: C23.0	NR	NR
Koppatz 2021 [15]	Southern Finland Regional Cancer Center (FICAN South)	Finland	GBC, ICD codes not reported	NR	NR
Koshiol 2022 [16]	NPCR-SEER database SEER-18	US	BTC NOS: C24.8, C24.9<br>AC: C24.1<br>iCCA: C22.0, C22.1<br>eCCA: C24.0 + Klatskin tumours[a]<br>GBC: C23.9	**C24.0** (8032/3, 8070/1, 8140, 8160, 8162/3, 8260, 8480, 8481, 8490, or 8560)<br>**C22.0** (8160/3)<br>**C22.1** (8032/3, 8070/1, 8140, 8160, 8260, 8480, 8481, 8490, or 8560)	**eCCA**<br>8160/0: Bile duct adenoma<br>8160/3: CCA (iCCA, eCCA)<br>8162/3: Perihilar CCA[a], Klatskin tumour[a]
Lee 2021 [17]	SEER-18	US	iCCA: C22.0, C22.1	C22.0, C22.1 (8160/3)	8160/3: CCA
Lin 2022 [18]	Taiwan cancer registry	Taiwan	iCCA: C22.1	Excluded 9590-9993	NR

Study	Study type/ data source	Study location	ICD-10/ICD-O-3 code(s)	Morphology codes	BTC-specific morphology codes
Makiuchi 2023 [19]	National Cancer Registry	Japan	AC: C24.1<br>iCCA: C22.1<br>eCCA: C24.0<br>GBC: C23.9	8260/3, 8211/3, 8160/3, 8161/3, 8170/3, 8180/3, 8140/3, 8480/3, 8490/3, 8560/3, 8033/3, 8070/3, 8020/3, 9100/3, 8980/3, 8240/3, 8249/3, 8246/3, 8013/3, 8041/3, 8244/3, 8470/3, 8503/3, 8000/3	8160/3: CCA<br>8161/3: Bile duct cystadenocarcinoma<br>8180/3: Combined hepatocellular carcinoma and CCA (iCCA, eCCA, GBC)
Mancini 2022 [20]	13 cancer registries in Italy	Italy	iCCA: C22.0	8013, 8020, 8041, 8154, 8160–8162, 8180, 8240, 8246, 8249, 8470	8160/0: Bile duct adenoma<br>8160/3: CCA<br>8161/0: Bile duct cystadenoma<br>8161/3: Bile duct cystadenocarcinoma<br>8162/3: Perihilar CCA[a], Klatskin tumour[a]<br>8180/3: Combined HCC and CCA
Miranda-Filho 2020 [21]	Data shown are for the US, SEER-18 database	Global	AC: C24.1<br>eCCA: C24.0<br>GBC: C23.0	NR	NR
Neuzillet 2022 [22]	PMSI database	France	iCCA: C22.1	NR	NR
Rahib 2021 [23]	SEER	US	Liver and intrahepatic bile duct, GBC, ICD codes not reported	NR	NR
Rahman 2022 [24]	Swedish National Cancer Register	Sweden	***Study used ICD-O-2 codes:***<br>BTC NOS: C24.8, C24.9 + Klatskin tumours[a]<br>iCCA: C22.0, C22.1<br>dCCA:[a] C24.1, C17.0<br>pCCA:[a] C24.0<br>GBC: C23.9	***Study used ICD-O-2 codes:***<br>**C24.8/C24.9** (8162, 8000)<br>**C22.0/C22.1** (8140, 8148)<br>**C24.1/C17.0** (8160)<br>**C24.0** (8180)<br>**C23.9** (8470, 8480)	Refer to ICD-O-2 morphology codes
Raza 2022 [25]	USCS registry (uses data from NPCR and SEER)	US	GBC: C23.9	NR	NR
Rumgay 2022 [26]	GLOBOCAN 2018	Global	iCCA: C22.0	(8050, 8140-8141, 8160-8161, 8260, 8440, 8480-8500, 8570-8572)	8160/0: Bile duct adenoma<br>8160/3: CCA<br>8161/0: Bile duct cystadenoma<br>8161/3: Bile duct cystadenocarcinoma
SEER [27]	SEER-22	US	iCCA: C22.1	NR	NR

Study	Study type/<br>data source	Study location	ICD-10/ICD-O-3 code(s)	Morphology codes	BTC-specific morphology codes
			GBC: C23.0		
Selvadurai 2021 [28]	3 regional hospitals in England	UK	AC: C24.1<br>iCCA: C22.1<br>eCCA: C24.0	NR	NR
Tella 2023 [29]	SEER-18	US	iCCA: C22.0, C22.1<br>eCCA: C22.0, C24.0	NR	NR
Velasco 2020 [30]	Girona Cancer Registry	Spain	BTC NOS: C24.9<br>AC: C24.1<br>iCCA: C22.1<br>eCCA: C24.0<br>GBC: C23.9	NR	NR
Xie 2022 [31]	Data from Global Burden of Disease Study 2019	US	BTC + GBC, ICD codes not reported	NR	NR
Xing 2022 [32]	SEER	US	iCCA: C22.1	(8160/3)	8160/3: CCA
Zhu 2020 [33]	SEER-18	US	iCCA: C22.1	(8010, 8020, 8140, 8160, 8161)	8160: Bile duct adenoma<br>8161/0: Bile duct cystadenoma<br>8161/3: Bile duct cystadenocarcinoma

AC, ampullary cancer; BTC, biliary tract cancer; CCA, cholangiocarcinoma; dCCA, distal cholangiocarcinoma; eCCA, extrahepatic cholangiocarcinoma; EHR, electronic health record; GBC, gallbladder cancer; HCC, hepatocellular carcinoma; iCCA, intrahepatic cholangiocarcinoma; ICD, International Classification of Diseases; ICD-O, International Classification of Diseases for Oncology; KCCR, Korea Central Cancer Registry; KKCR, Khon Kaen Cancer Registry; N/A, not applicable; NOS, not otherwise specified; NPCR, National Program of Cancer Registries; NR, not reported; pCCA, perihilar cholangiocarcinoma; PSMI, Programme de médicalisation des systèmes d’information; SEER, Surveillance, Epidemiology, and End Results; UK, United Kingdom; US, United States; USCS, United States Cancer Statistics

[a] dCCA and pCCA, also called hCCA or ‘Klatskin tumours’, are subtypes of eCCA

Table S2: Characteristics of studies included in the review of the frequency of MDM2 amplifications

Study	Country	Population, N	Cancer site	Definition of MDM2 amplification	Assessment method	Proportion of patients with MDM2 amplification
Battaglin 2020 [34]	NR	N=1,860	BTC	Copy number ≥6	DNA sequencing data (NGS)	3.4% (64/1,860)
Bouattour 2023 (TOPAZ-1 trial; NCT03875235) [35]	International	Biomarker-evaluable population N=441 (n=214 receiving durvalumab + GemCis; n=227 receiving placebo + GemCis)	BTC, no prior treatment	NR	NR	8.2% (36/441)
Cassier 2017 [36]	NR	BTC N=45<br>(iCCA n=32; vesicular carcinoma n=7; main biliary duct carcinoma n=5; unknown n=1)	BTC, iCCA, vesicular carcinoma, main biliary duct carcinoma	NR	DNA sequencing data (NGS), whole genome array comparative genomic hybridization<br>(34 pts successfully assessed with at least one of the 2 methods)	8.8% (3/34)
D'Afonseca 2020 [37]	Global	Patients, N=133 (tissue samples N=135) from 2 repositories<br>Repository 1:<br>US, n=49<br>Chile, n=21<br>Japan, n=11<br>Others, n=20<br>Repository 2 (no information on CNAs)<br>China, n=32	GBC	NR	Data from public repositories (DNA sequencing data)<br>Repository 1: targeted sequencing samples<br>Repository 2: data from CBioPortal; does not contain information on CNAs	13.2% (sample size on which information is based is not specified; most MDM2 amplifications found in samples from the US)
Feng 2021 [38]	China	63 patient samples collected; mutations detected in 60 samples	hCCA[e]	NR	DNA sequencing data (NGS based YuanSu450™ gene panel of OrigiMed [Shanghai, China]; CLIA and CAP certified laboratories)<br>Copy-number variation regions identified by Control-FREEC (v9.7) with the following parameters:	10.0% (6/60)

Study	Country	Population, N	Cancer site	Definition of MDM2 amplification	Assessment method	Proportion of patients with MDM2 amplification
					window=50 000 and step=10 000	
Harthimmer 2019 [39]	Denmark	59 patient samples collected; mutational analysis of 54 samples	AC	NR	DNA sequencing data (NGS); hybrid capture based NGS platform (Illumina HiSeq2500 instrument (Illumina, San Diego, California US)	7.4%; (CI: 2-18) (4/54)
Kato 2018 [40]	NR	GBC n=554 (GENIE project GBC n=70)<br><br>Bile duct adenocarcinoma n=248	GBC, bile duct adenocarcinoma	≥8 copies above ploidy, with ≥6 copies considered equivocal	NGS - Foundation Medicine, Cambridge, MA	GBC: 11.19% (62/554)<br>(GENIE project GBC: 17.5%; 7/40)<br>Bile duct adenocarcinoma: 5.24% (13/248)
Kendre 2023 [41]	NR	N=6,130	iCCA	NR	DNA sequencing data (Hybrid capture-based comprehensive genomic profiling) FoundationOne® (F1) and FoundationOne® CDx (F1CDx) assays in a CLIA certified and CAP accredited laboratory	4.3% (261/6,130)
Kim 2018 [42]	Japan and Korea	GBC: N=216<br>iCCA: N=213<br>Large duct n=110<br>Small duct n=103<br>eCCA: N=133<br>hCCA[e] n=68<br>dCCA[e] n=65	GBC<br>iCCA, eCCA<br>(hCCA[e], dCCA[e])	Ratio of MDM2:CHR12 counted in 40 tumour cells in each case; average ratios >2.0 were considered to be amplified	DISH on tissue microarray sections using an automated staining platform (Ventana BenchMark XT system; Ventana Medical Systems, Tucson, AZ)	GBC: 14% (30/216)<br>iCCA: 6% (13/213)<br>large duct: 12% (13/110)<br>small duct: 0%<br>eCCA: 6% (8/133)<br>hCCA:[e] 12% (8/68)<br>dCCA: [e] 0%
Kumar-Sinha 2023 [43]	Italy	N=124	BTC	NR	Illumina HiSeq 2000 or HiSeq 2500: n=122<br>commercial sequencing assays n=32	4% (5/124)
Lee 2016 [44]	US	N=99	eCCA	NR	Hybrid capture-based comprehensive genomic profiling in CLIA certified and CAP accredited laboratories	5.0% (5/99)

Study	Country	Population, N	Cancer site	Definition of MDM2 amplification	Assessment method	Proportion of patients with MDM2 amplification
Lin 2020 [45]	NR	N=803	BTC	NR	DNA sequencing data (whole exon sequencing [160 samples] and hybrid capture-based comprehensive genomic profiling [643 samples])	6.6% (53/803; no data on CN calls are available)
Lin 2024 [46]	US	iCCA N= 327<br>eCCA N=62<br>hCCA[e] n=30<br>GBC N=64	CCA (iCCA, eCCA, hCCA[e]), GBC	NR	DNA sequencing data (NGS; Foundation One® CDx)	iCCA: 2.8% (9/327)<br>eCCA: 6.5% (4/62)<br>hCCA:[e] 13.3% (4/30)<br>GBC: 10.9% (7/64)
MyCancer Genome [47]	NR	NR	CCA<br>AC	NR	NR	CCA: 4.35%<br>AC: 8.97%
Pu 2020 [48]	Spain, China	N=132 (Spain; from database) 140 (China, tissue samples)	iCCA	Fluorescent in situ hybridization: amplification was defined at target/contrast signals ratio >2 and the average target signal ≥6	DNA sequencing (NGS) of samples from Spain (MSK-IMPACT platform); fluorescent in situ hybridization for samples from China	Spanish cohort: 2.27% (3/132)<br>Chinese cohort: 5.71% (8/140)
Rimini 2023 [49]	Italy	N=51	BTC	NR	324-gene NGS panel	10% (5/51)
Simbolo 2019 [50]	NR	eCCA N= 99<br>dCCA[e] n=52<br>pCCA[e] n=47	eCCA (dCCA[e], pCCA[e])	NR	High-coverage targeted sequencing, confirmation of copy number gains by quantitative PCR and fluorescent in situ hybridization	NR
Wong 2019 [51]	US	N=45	AC	NR	DNA sequencing data (NGS, MSK-IMPACT)	16% (7/45)[d]
Zheng 2021 [52]	China	CCA N=270<br>iCCA n= 92<br>pCCA[e] n=70<br>dCCA[e] n=108	CCA (iCCA, pCCA[e], dCCA[e])	NR	DNA sequencing data (NGS based YuanSu450™ gene panel of OrigiMed [Shanghai, China]; CLIA and CAP certified laboratories; Illumina NextSeq-500)	CCA: 3.7% (10/270)<br>iCCA: 1.1% (1/92)<br>eCCA: NR, calculated as 5.6% (10/178)<br>pCCA:[de] 8.6% (6/70)<br>dCCA:[e] 3.7% (4/108)

BTC, biliary tract cancer; CAP, College of American Pathologists; CCA, cholangiocarcinoma; CHR12, chromosome 12; CLIA, Clinical Laboratory Improvement Amendments; CN, copy number; CNA, copy number alteration; dCCA, distal cholangiocarcinoma; DISH, dual-colour in situ hybridization; DNA, deoxyribonucleic acid; GBC, gallbladder cancer; GemCis, gemcitabine + cisplatin; hCCA, hilar cholangiocarcinoma; iCCA, intrahepatic cholangiocarcinoma; IQR, interquartile

range; MDM2, mouse double minute 2 homolog; MSK-IMPACT, Memorial Sloan Kettering - Integrated Mutation Profiling of Actionable Cancer Targets; NGS, next generation sequencing; NR, not reported; pCCA, perihilar cholangiocarcinoma; pts, patients; US, United States

[a] In TOPAZ-1, the long-term survivor population included patients who survived ≥18 months; the non-long term survivor population included patients who survived <18 months.

[b] Among long-term survivors, MDM2 amplification was present in 6.0% of 67 patients in the durvalumab + GemCis group and 17% of 48 patients in the placebo + GemCis group

[c] Among non-long-term survivors, MDM2 amplification was present in 8.2% of patients in the durvalumab + GemCis group and 6.7% of 179 patients in the placebo + GemCis group

[d] Value calculated for proportion of samples with MDM2 amplification only, excluding samples with other MDM2 gene alterations

[e] dCCA and pCCA, also called hCCA or 'Klatskin tumours', are subtypes of eCCA

Table S3: Global studies: Incidence of BTC

Study	Country, database/ registry	Year(s)	Cancer site, definition[a]	ASR/100,000 (both sexes)[b]	ASR/100,000 (males)[b]	ASR/100,000 (females)[b]	New cases/year
Baria 2022 [4],[c]	**Global**	2008-2012	BTC, iCCA, eCCA, GBC, AC	**BTC**<br>**Global range**: **1.12-12.42**<br>**Country data**:<br>China: 3.26<br>Japan: 5.78<br>South Korea: 9.00<br>Thailand: 4.23<br>**eCCA**<br>**Global range: 0.10-2.71**<br>**iCCA**<br>**Global range: 0.16-2.18**<br>**GBC**<br>**Global range: 0.39-9.68**<br>**AC**<br>**Global range: 0.18-0.93**	**Asia:**<br>China: eCCA 1.01; iCCA 0.70; GBC 1.07; AC 0.27<br>Japan: eCCA 3.68; iCCA 0.90; GBC 1.96; AC 0.61<br>South Korea: eCCA 3.81; iCCA 3.10; GBC 2.99; AC 1.16<br>Thailand: eCCA 1.50; iCCA 2.26; GBC 0.71; AC 0.49<br>**EU-4 and the UK:**<br>France: eCCA 0.80; iCCA 1.32; GBC 0.45; AC 0.52<br>Germany: eCCA 0.98; iCCA 0.80; GBC 0.48; AC 0.53<br>Italy: eCCA 1.06; iCCA 0.88; GBC 0.81; AC 0.50<br>Spain: eCCA 0.89; iCCA 0.88; GBC 0.66; AC 0.63<br>UK: eCCA 0.37; iCCA 0.76; GBC 0.34; AC 0.42<br>**US:**<br>US: eCCA 0.68; iCCA 0.72; GBC 0.47; AC 0.40	**Asia:**<br>China: eCCA 0.85; iCCA 0.47; GBC 1.63; AC 0.19<br>Japan: eCCA 1.01; iCCA 0.70; GBC 1.07; AC 0.32<br>South Korea: eCCA 1.89; iCCA 1.41; GBC 2.83; AC 0.75<br>Thailand: eCCA 0.72; iCCA 1.28; GBC 1.03; AC 0.39<br>**EU-4 and the UK:**<br>France: eCCA 0.42; iCCA 0.76; GBC 0.64; AC 0.34<br>Germany: eCCA 0.56; iCCA 0.59; GBC 0.85; AC 0.31<br>Italy: eCCA 0.73; iCCA 0.59; GBC 1.23; AC 0.30<br>Spain: eCCA 0.49; iCCA 0.57; GBC 0.96; AC 0.30<br>UK: eCCA 0.31; iCCA 0.69; GBC 0.68; AC 0.28<br>**US:**<br>US: eCCA 0.47; iCCA 0.57; GBC 0.82; AC 0.25	NR

Florio 2020 [8],c	Global	2008-2012	iCCA, eCCA C22, C24.0	**iCCA Global range: 0.26–2.80** **<u>Asia:</u>** China: 0.63 (0.57, 0.69) Japan: 0.95 (0.89, 1.00) South Korea: 2.80 (2.68, 2.93) Thailand: 2.19 (2.01, 2.36) **<u>EU-4, the UK and Nordics:</u>** Denmark: 0.60 (0.53, 0.67) Norway: 0.45 (0.38, 0.52) France: 1.13 (1.02, 1.23) UK: 1.15 (1.12, 1.18) Italy: 0.58 (0.48, 0.69)	**eCCA Global range: 0.08–2.24** **<u>Asia:</u>** China: 0.55 (0.49, 0.61) Japan: 0.83 (0.78, 0.88) South Korea: 2.24 (2.13, 2.35) Thailand: 0.71 (0.60, 0.82) **<u>EU-4, the UK and Nordics:</u>** Denmark: NR Norway: 0.44 (0.38, 0.51) France: 0.39 (0.33, 0.45) UK: 0.29 (0.27, 0.30) Italy: 0.48 (0.39, 0.57) Spain: 0.49 (0.43,	**iCCA Global range: 0.27–3.80** **<u>Asia:</u>** China: 0.69 (0.59, 0.78) Japan: 1.29 (1.19, 1.38) South Korea: 3.80 (3.58, 4.02) Thailand: 2.93 (2.63, 3.22) **<u>EU-4, the UK and Nordics:</u>** Denmark: 0.62 (0.52, 0.72) Norway: 0.50 (0.40, 0.60) France: 1.41 (1.24, 1.59) UK: 1.26 (1.21, 1.30)	**eCCA Global range: 0.08–3.36** **<u>Asia:</u>** China: 0.56 (0.47, 0.65) Japan: 1.15 (1.06, 1.24) South Korea: 3.36 (3.15, 3.57) Thailand: 0.97 (0.80, 1.14) **<u>EU-4, the UK and Nordics:</u>** Denmark: NR Norway: 0.52 (0.41, 0.62) France: 0.55 (0.44, 0.66) UK: 0.32 (0.29, 0.34) Italy: 0.60 (0.45, 0.74)	**iCCA Global range: 0.24–1.99** **<u>Asia:</u>** China: 0.58 (0.49, 0.67) Japan: 0.67 (0.60, 0.74) South Korea: 1.99 (1.85, 2.14) Thailand: 1.54 (1.34, 1.74) **<u>EU-4, the UK and Nordics:</u>** Denmark: 0.60 (0.50, 0.69) Norway: 0.39 (0.31, 0.48) France: 0.88 (0.75, 1.02) UK: 1.07 (1.03, 1.11)	**eCCA Global range: 0.07–1.36** **<u>Asia:</u>** China: 0.52 (0.44, 0.61) Japan: 0.57 (0.51, 0.63) South Korea: 1.36 (1.24, 1.47) Thailand: 0.48 (0.35, 0.61) **<u>EU-4, the UK and Nordics:</u>** Denmark: NR Norway: 0.37 (0.29, 0.46) France: 0.25 (0.18, 0.31) UK: 0.26 (0.24, 0.28) Italy: 0.38 (0.26, 0.49)	**iCCA (both sexes):** China: 441 Japan: 1,291 South Korea: 1,939 Thailand: 753 Denmark: 316 Norway: 201 France: 503 UK: 6,959 Italy: 153 Spain: 346 Germany: 121 US: 1,881	**eCCA (both sexes)** China: 418 Japan: 1,234 South Korea: 1,548 Thailand: 226 Denmark: NR Norway: 192 France: 188 UK: 1,630 Italy: 135 Spain: 261 Germany: 94 US: 1,494

Study	Country, database/ registry	Year(s)	Cancer site, definition[a]	ASR/100,000 (both sexes)[b]	ASR/100,000 (males)[b]	ASR/100,000 (females)[b]	New cases/year
				Spain: 0.74 (0.66, 0.83)<br>Germany: 1.05 (0.84, 1.26)<br>**US:**<br>US: 0.78 (0.74, 0.82)<br>0.56)<br>Germany: 0.74 (0.57, 0.91)<br>**US:**<br>US: 0.58 (0.54, 0.61)	Italy: 0.71 (0.54, 0.87)<br>Spain: 0.98 (0.84, 1.12)<br>Germany: 1.26 (0.93, 1.59)<br>**US:**<br>US: 0.88 (0.82, 0.94)<br>Spain: 0.74 (0.62, 0.85)<br>Germany: 1.03 (0.74, 1.32)<br>**US:**<br>US: 0.71 (0.66, 0.76)	Italy: 0.48 (0.35, 0.61)<br>Spain: 0.52 (0.43, 0.62)<br>Germany: 0.86 (0.60, 1.12)<br>**US:**<br>US: 0.69 (0.65, 0.74)<br>Spain: 0.28 (0.21, 0.35)<br>Germany: 0.49 (0.31, 0.67)<br>**US:**<br>US: 0.46 (0.42, 0.50)	
Rumgay 2022 [26][c]	GLOBOCAN	2018	**iCCA**<br>ICD-O-3 C22	**Global average: 1.4**<br>**Global range: 0.2-8.1**<br>China: 2.1<br>Japan: 0.6<br>South Korea: 3.3<br>Thailand: 5.2<br>Denmark: 1.3<br>Finland: 1.1<br>Norway: 0.9<br>Sweden: 1.4<br>UK: 2.2<br>Italy: 1.1<br>Spain: 1.4<br>France: 1.6<br>Germany: 1.0<br>US: 1.1	**Global average: 1.6**<br>**Global Range: 0.0-8.5**<br>China: 2.5<br>Japan: 0.9<br>South Korea: 4.3<br>Thailand: 6.1<br>Denmark: 1.4<br>Finland: 1.2<br>Norway: 1.2<br>Sweden: 1.7<br>UK: 2.2<br>Italy: 1.5<br>Spain: 1.8<br>France: 2.2<br>Germany: 1.1<br>US: 1.2	**Global average: 1.2**<br>**Global range: 0.1-7.7**<br>China: 1.6<br>Japan: 0.4<br>South Korea: 2.4<br>Thailand: 4.4<br>Denmark: 1.2<br>Finland: 1.0<br>Norway: 0.6<br>Sweden: 1.1<br>UK: 2.1<br>Italy: 0.8<br>Spain: 1.0<br>France: 1.1<br>Germany: 0.8<br>US: 1.0	**Both sexes:** 123,000<br>**Males:** 67,000<br>**Females:** 56,000
Xie 2022 [31]	Global, data from Global Burden of Disease Study 2019	2019	**BTC+GBC**	2.5 (2.1, 2.7)	2.4 (1.9, 2.7)	2.6 (2.1, 3.0)	**Both sexes:** 199,200<br>**Males:** 86,400<br>**Females:** 112,800

GLOBOCAN [10]	Global data, GLOBOCAN	2020	**GBC** C23	**Global: 1.2**<br>**Global range: 0.03–8.5**<br>**<u>Asia:</u>**<br>China: 1.2<br>Japan: 1.8<br>South Korea: 2.9<br>Thailand: 1.5<br>**<u>EU-5 and Nordics:</u>**<br>Denmark: 0.41<br>Finland: 0.84<br>Norway: 0.39<br>Sweden: 0.89<br>UK: 0.71<br>Italy: 0.55<br>Spain: 0.49<br>France: 0.39<br>Germany: 0.52<br>**<u>US:</u>**<br>US: 0.68	**Global: 0.89**<br>**Global range: 0.06–7.3**<br>**<u>Asia:</u>**<br>China: 0.98<br>Japan: 1.8<br>South Korea: 3.2<br>Thailand: 1.8<br>**<u>EU-5 and Nordics:</u>**<br>Denmark: 0.37<br>Finland: 0.73<br>Norway: 0.36<br>Sweden: 0.92<br>UK: 0.41<br>Italy: 0.49<br>Spain: 0.44<br>France: 0.33<br>Germany: 0.39<br>**<u>US:</u>**<br>US: 0.51	**Global: 1.4**<br>**Global range: 0.04–9.6**<br>**<u>Asia:</u>**<br>China: 1.4<br>Japan: 1.8<br>South Korea: 2.8<br>Thailand: 1.2<br>**<u>EU-5 and Nordics:</u>**<br>Denmark: 0.45<br>Finland: 0.95<br>Norway: 0.41<br>Sweden: 0.85<br>UK: 0.98<br>Italy: 0.61<br>Spain: 0.53<br>France: 0.44<br>Germany: 0.63<br>**<u>US:</u>**<br>US: 0.82	**<u>Asia (both sexes):</u>**<br>China: 28,923<br>Japan: 9,734<br>South Korea: 3,437<br>Thailand: 1,883<br>**<u>EU-5 and Nordics (both sexes):</u>**<br>Denmark: 60<br>Finland: 137<br>Norway: 49<br>Sweden: 217<br>UK: 1,238<br>Italy: 1,071<br>Spain: 721<br>France: 655<br>Germany: 1,390<br>**<u>US (both sexes):</u>**<br>US: 4,670<br>**<u>Asia (males):</u>**<br>China: 11,061<br>Japan: 4,013<br>South Korea: 1,598<br>Thailand: 993<br>**<u>EU-5 and Nordics (males):</u>**<br>Denmark: 24<br>Finland: 55<br>Norway: 20<br>Sweden: 94<br>UK: 348<br>Italy: 402<br>Spain: 276<br>France: 232<br>Germany: 445<br>**<u>US (males):</u>**<br>US: 1,626<br>**<u>Asia (females):</u>**

Study	Country, database/ registry	Year(s)	Cancer site, definition[a]	ASR/100,000 (both sexes)[b]	ASR/100,000 (males)[b]	ASR/100,000 (females)[b]	New cases/year
							China: 17,862<br>Japan: 5,721<br>South Korea: 1,839<br>Thailand: 890<br>**EU-5 and Nordics (females):**<br>Denmark: 36<br>Finland: 82<br>Norway: 29<br>Sweden: 123<br>UK: 890<br>Italy: 669<br>Spain: 445<br>France: 423<br>Germany: 945<br>**US (females):**<br>US: 3,044

ASR, age standardised rate; BTC, biliary tract cancer; eCCA, extrahepatic cholangiocarcinoma; GBC, gallbladder cancer; GLOBOCAN, Global Cancer Observatory; iCCA, intrahepatic cholangiocarcinoma; ICD (O), International Classification of Diseases (Oncology); NR, not reported; UK, United Kingdom; US, United States

[a] ICD-O-3 morphology codes for each study are provided in Table S1. [b] All numbers in parentheses are 95% confidence intervals unless otherwise stated.
[c] Studies reporting 'global data' differed in the number of countries investigated and/or reported. Baria et al., 2022 evaluated data from 65 countries and reported data for 22 countries. Florio et al., 2020 reported data from 38 and 33 countries for iCCA and eCCA, respectively, using either national data or aggregated regional data. Rumgay et al., 2022 used 2018 data from IARC (GLOBOCAN) for 185 countries.

Table S4: United States: Incidence of BTC

US Study/database	Dataset/registry	Year(s)	Cancer site, definition[a]	ASR/100,000 (both sexes)[b]	ASR/100,000 (males)[b]	ASR/100,000 (females)[b]	Incidence, new cases/year
SEER [27]	US, SEER-22	2020	Liver and intrahepatic bile duct cancer<br>C220, C221	8.5 (8.3, 8.6)	12.6 (12.4, 12.9)	4.9 (4.8, 5.0)	**Both sexes:** 16,596<br>**Males:** 11,497<br>**Females:** 5,099
SEER [27]	US, SEER-22	2020	GBC<br>C23.9	1.1 (1.1, 1.2)	0.9 (0.8, 0.9)	1.4 (1.3, 1.4)	**Both sexes:** 2,099<br>**Males:** 717<br>**Females:** 1,382
Ali 2022 [1]	US, SEER-18	2000-2017	iCCA<br>ICD-0-3/WHO 2008: C22.1	Diagnoses in 2012 and beyond: 1.1	2001-2017: 1.1	2001-2017: 0.8	13,886 pts diagnosed 2000-2017
Alkhayyat 2021 [2]	US, 'Explorys'<br>EHR data (26 health systems across the US)	2018-2019	GBC<br>SNOMED-CT: 'Primary malignant neoplasm of gallbladder'	7.4	NR	NR	NR
Javle 2022 [12]	US, SEER-18	2001-2017	CCA<br>iCCA: C22.1<br>eCCA: C24.0, C24.1, C24.9	2001-2017<br>**CCA**: 3.65<br>**iCCA**: 1.19<br>**eCCA**: 2.46<br>2017<br>**CCA**:4.43<br>**iCCA:** 1.99<br>**eCCA**: 2.45	2001-2017<br>**CCA**: 4.40<br>2017<br>**CCA**: 5.35	2001-2017<br>**CCA**: 3.08<br>2017<br>**CCA**: 3.68	NR

US Study/database	Dataset/registry	Year(s)	Cancer site, definition[a]	ASR/100,000 (both sexes)[b]	ASR/100,000 (males)[b]	ASR/100,000 (females)[b]	Incidence, new cases/year
Miranda-Filho 2020 [21]	Global study focused on the Americas. Data shown are for the US, SEER-18 database	2008-2012	GBC: C23<br>Extrahepatic duct: C24<br>Ampulla of Vater: C24.1	NR	**GBC:** Variation according to race/ethnicity: 0.40-0.74<br>**eCCA:** Variation according to race/ethnicity: 0.49-0.87<br>**AC:** Variation according to race/ethnicity: 0.18-0.67	**GBC:** Variation according to race/ethnicity: 0.64-1.78<br>**eCCA:** Variation according to race/ethnicity: 0.40-0.73<br>**AC:** Variation according to race/ethnicity: 0.18-0.43	**GBC females**: 104-2,657<br>**GBC males:** 57-1,187<br>**eCCA females:** 40-1,585<br>**eCCA males:** 40-1,912<br>**AC females:** 19-818<br>**AC males:** 17-1,519
Koshiol 2022 [16]	US, NPCR-SEER database<br>SEER-18 (18 registries)	2001-2015	BTC<br>eCCA: C24.0<br>iCCA: C22.1; C22.0<br>GBC: C23.9<br>AC: C24.1<br>Klatskin tumours[c]	**BTC:** 5.04 (5.02, 5.06)<br>**GBC:** 1.57 (1.56, 1.58)<br>**iCCA:** 1.17 (1.16, 1.19)<br>**eCCA:** 1.25 (1.24, 1.26)<br>**AC:** 0.76 (0.75, 0.77)	**BTC:** 5.31 (5.27, 5.34)<br>**GBC:** 1.14 (1.12, 1.16)<br>**iCCA:** 1.33 (1.31, 1.35)<br>**eCCA:** 1.55 (1.53, 1.57)<br>**AC:** 0.97 (0.95, 0.98)	**BTC:** 4.85 (4.82, 4.89)<br>**GBC:** 1.92 (1.90, 1.94)<br>**iCCA:** 1.05 (1.04, 1.07)<br>**eCCA:** 1.02 (1.01, 1.04)<br>**AC:** 0.61 (0.60, 0.62)	NR
Lee 2021 [17]	US, SEER-18	2000-2017	iCCA<br>C22.0, C22.1	NR	NR	NR	13,611 pts diagnosed 2000-2017

US Study/database	Dataset/registry	Year(s)	Cancer site, definition[a]	ASR/100,000 (both sexes)[b]	ASR/100,000 (males)[b]	ASR/100,000 (females)[b]	Incidence, new cases/year
Rahib 2021 [23]	US, SEER (2014-2016 data) with existing US Census Bureau demographic projections based on 2010 US Census data	Projected to 2040	Liver and intrahepatic bile duct cancer	NR	NR	NR	**Both sexes:**<br>2020:43,000<br>2030: 68,000<br>2040: 100,000<br>**Males:**<br>2020: 31,000<br>2030: 46,000<br>2040: 65,000<br>**Females:**<br>2020: 12,000<br>2030: 22,000<br>2040: 34,000
Raza 2022 [25]	US, USCS registry (uses data from NPCR and SEER)	2001-2018	GBC<br>C23.9	<u>2018:</u><br>1.54 (1.49, 1.59)<br><u>2001-2018:</u><br>1.59 (1.58, 1.60)	<u>2001-2018:</u><br>0.82 (0.81, 0.84)	<u>2001-2018:</u><br>1.40 (1.37, 1.41)	<u>2018:</u> 4,342<br><br>68,206 pts diagnosed 2001-2018
Tella 2023 [29]	US, SEER-18	2000-2017	eCCA, iCCA<br>C220, C21, C240	**iCCA**: 1.07<br>**eCCA**: 0.74<br><br>Predicted incidence (ASR, 2018-2029):<br>**iCCA**: 2.13<br>**eCCA**:1.01	NR	NR	12,737 pts with iCCA and 8,395 pts with eCCA diagnosed 2000-2017
Xing 2022 [32]	US, SEER	2000-2018	iCCA<br>C22.1	2000: 0.6<br>2018: 1.3	NR	NR	2,516 pts 2010-2015
Zhu 2020 [33]	US, SEER-18	2011-2015	iCCA<br>C22.0 or C22.1	NR	1.49	1.20	3,456 pts 2011-2015

AC, ampullary cancer; ASR, age standardised rate; BTC, biliary tract cancer; CCA, cholangiocarcinoma; eCCA, extrahepatic cholangiocarcinoma; EHR, electronic health record; GBC, gallbladder cancer; GLOBOCAN, Global Cancer Observatory; iCCA, intrahepatic cholangiocarcinoma; ICD (O), International Classification of Diseases (Oncology); NPCR, National Program of Cancer Registries; NR, not reported; pts, patients; SEER, Surveillance, Epidemiology, and End Results; US, United States; USCS, US Cancer Statistics; WHO, World Health Organization

[a] ICD-O-3 morphology codes for each study are provided in Table S1. [b] All numbers in parentheses are 95% confidence intervals unless otherwise stated.
[c] 'Klatskin tumours', also called hCCA or pCCA, are subtypes of eCCA.

Table S5: EU-4, the UK and Nordics: Incidence of BTC

Study/database	Dataset/registry	Year(s)	Cancer site, definition[a]	ASR/100,000 (both sexes)[b]	ASR/100,000 (males)[b]	ASR/100,000 (females)[b]	Incidence, new cases/year
Ghiringhelli 2023 [9]	France, Digestive Cancer Registry of Burgundy	2012-2019	iCCA: C22.1, GBC: C23, eCCA: C24.0 AC: C24.1	NR	2018-2019 **iCCA**: 1.59 **GBC:** 0.43 **eCCA**: 0.94 **AC**: 0.36	NR	NR
Neuzillet 2022 [22]	France, PMSI database	2014-2015	iCCA C22.1	NR	NR	NR	1825
Mancini 2022 [20]	Italy, 13 cancer registries	2003-2017	iCCA C22	NR	2003-2017: 1.8 (1.7, 1.9) 2013-2017: 2.2 (2.0, 2.3)	2003-2017: 1.1 (1.0, 1.2) 2013-2017: 1.2 (1.1, 1.4)	NR
Velasco 2020 [30]	Spain, Girona Cancer Registry	1994-2016	BTC iCCA: C22.1 GBC: C23.9 eCCA: C24.0 AC: C24.1 Overlapping lesion of biliary tract: C24.8 Biliary tract NOS: C24.9	BTC: ASRE13[c] (adjusted to European standard population): 8.39 (7.9, 8.9) BTC: ASRW[c] (adjusted to European standard population): 3.26 (3.0, 3.5)	NR	NR	1,102 pts diagnosed 1994-2016 eCCA: 26.2% GBC: 23.2% iCCA: 22.7% AC: 12.9% Overlapping and NOS: 14.8%
Selvadurai 2021 [28]	UK, 3 regional hospitals in England	2015-2017	iCCA: C22.1 eCCA: C24.0 GBC: C23X AC: C24.1	NR	NR	NR	15.7% iCCA; 1.4% eCCA; 11.7% GBC; 0.5% AC (N=625)

Study/database	Dataset/registry	Year(s)	Cancer site, definition[a]	ASR/100,000 (both sexes)[b]	ASR/100,000 (males)[b]	ASR/100,000 (females)[b]	Incidence, new cases/year
Barner-Rasmussen 2021 [5]	Finland, Finnish Cancer Registry (countrywide)	1978-2017 (CCA) 2013-2017 (iCCA, eCCA)	CCA, iCCA, eCCA<br>C22.1, C24.0, C24.8, C24.9	NR	1978-2017<br>**CCA**: 1.8 (1.5, 2.1)<br><br>2013-2017<br>**iCCA:** 1.3 (1.1, 1.5)<br>**eCCA**: 0.4 (0.3, 0.5)	1978-2017<br>**CCA**: 1.5 (1.3, 1.7)<br><br>2013-2017<br>**iCCA**: 1.0 (0.8, 1.2)<br>**eCCA**: 0.3 (0.2, 0.4)	NR
Koppatz 2021 [15]	Finland, Southern Finland Regional Cancer Center (FICAN South)	2006-2017	GBC (patients with other BTC were excluded)	2014-2017:<br>2.33 (1.88, 2.86)	NR	NR	24.5
Rahman 2022 [24]	Sweden, Swedish National Cancer Register	2011-2019	iCCA: C22.0/22.1<br>GBC: 23.9<br>pCCA[a] C24.0<br>dCCA[a] C24.1/17.0<br>NOS C24.8/24.9 incl.<br>Klatskin tumours[a] (ICD-O-2)	**BTC**: 8.39 (6.69, 10.51)<br>**iCCA:** 2.33 (1.52, 3.58)<br>**GBC**: 2.17 (1.39, 3.38)<br>**pCCA**:[a] 1.80 (1.10, 2.93)<br>**dCCA**:[a] 1.53 (0.91, 2.60)	NR	NR	NR

AC, ampullary cancer; ASR, age standardised rate; BTC, biliary tract cancer; CCA, cholangiocarcinoma; dCCA, distal cholangiocarcinoma; eCCA, extrahepatic cholangiocarcinoma; EU, European Union; GBC, gallbladder cancer; GLOBOCAN, Global Cancer Observatory; iCCA, intrahepatic cholangiocarcinoma; ICD (O), International Classification of Diseases (Oncology); NR, not reported; NOS, not otherwise specified; pCCA, perihilar cholangiocarcinoma; PMSI, Programme de Médicalisation des Systèmes d'Information; UK, United Kingdom

[a] ICD-O-3 morphology codes for each study are provided in Table S1. [b] All numbers in parentheses are 95% confidence intervals unless otherwise stated.
[c] Incidence rates are age-adjusted to the European standard population (ASRE) and the world standard population (ASRW). [d] dCCA and pCCA, also called hCCA or 'Klatskin tumours', are subtypes of eCCA.

Table S6: Asia: Incidence of BTC

Study/database	Dataset/registry	Year(s)	Cancer site/definition[a]	ASR/100, 000 (both sexes)[b]	ASR/100, 000 (males)[b]	ASR/100, 000 (females)[b]	Incidence, new cases/year [b]
Chen 2022 [7]	China, Global Burden of Disease (GBD) Study 2019 (data for mainland China)	2019	BTC+GBC	2.01 (1.41, 2.41)	2.25 (1.52, 2.79)	1.84 (1.10, 2.41)	**Both sexes:** 38, 634 (27, 350, 46, 512) **Males:** 19, 761 (13, 243, 24, 912) **Females:** 18, 872 (11, 321, 24, 679)
Makiuchi 2023 [19]	Japan, National Cancer Registry	2016-2017	iCCA: C22.1 GBC: C23.9 eCCA: C24.0 AC: C24.1	NR	**iCCA:** 2.8 **GBC**: 2.5 **eCCA**: 5.1 **AC:** 1.0	**iCCA:** 1.4 **GBC:** 2.4 **eCCA**: 2.2 **AC:** 0.6	NR
Hong 2022 [11]	KCCR based on the Korea National Cancer Incidence Database	1999-2019	iCCA C22.1	2019: 2.7	2019: 3.7	NR	2019: 2, 883
Kang 2022 [14]	Korea, KCCR based on the Korea National Cancer Incidence Database	2019	GBC: C23 eCCA: C24	**GBC**: 2.4 **eCCA**: 4.1	NR	NR	**GBC**: 2, 744 **eCCA**: 4, 639
Lin 2022 [18]	Taiwan, TCR	2008-2017	iCCA ICD-O-FT: T-155.1 before 2002 and ICD-O-3: C22.1	NR	2013-2017: 4.07	2008-2012: 2.95	Male/female incidence ratio 2013-2017: 1.52

Study/database	Dataset/registry	Year(s)	Cancer site/definition[a]	ASR/100, 000 (both sexes)[b]	ASR/100, 000 (males)[b]	ASR/100, 000 (females)[b]	Incidence, new cases/year [b]
Kamsa-Ard 2021 [13]	Thailand, KKCR, Northeast Thailand	1989-2018	CCA<br>C22.1, C24.0, C24.8, C24.9 (C24.1 - AC - was excluded)	NR	36.1 (35.3, 36.8)	14.4 (13.9, 14.8)	**Males:** 9, 426<br>**Females:** 4, 372

AC, ampullary cancer; ASR, age standardised rate; BTC, biliary tract cancer; CCA, cholangiocarcinoma; eCCA, extrahepatic cholangiocarcinoma; GBC, gallbladder cancer; GLOBOCAN, Global Cancer Observatory; iCCA, intrahepatic cholangiocarcinoma; ICD (O) (FT), International Classification of Diseases (Oncology) (Field Trial Edition); KCCR, Korea Central Cancer Registry; KKCR, Khon Kaen Cancer Registry; NR, not reported; TCR, Taiwan Cancer Registry

[a] ICD-O-3 morphology codes for each study are provided in Table S1. [b] All numbers in parentheses are 95% confidence intervals unless otherwise stated.

Table S7: Trends in incidence of BTC over time according to primary site

Study/database	Dataset/registry	Year(s)	Cancer site	Definition[a]	Estimated APC in incidence, % (95% CI)					
Florio 2020 [8]	Global	1993-2012	iCCA, eCCA	ICD-10: C22 and C24.0	**iCCA (both sexes):**<br>**Asia:**<br>China: 11.1 (−4.3, 28.9)<br>Japan: −0.7 (−5.5, 4.4)<br>South Korea: 4.5 (3.0, 5.9)<br>Thailand: −1.0 (−3.8, 1.9)<br>**EU-5 and Nordics:**<br>Denmark: −1.0 (−5.1, 3.2)<br>Norway: 5.2 (1.0, 9.5)<br>France: 6.5 (0.7, 12.5)<br>UK: 4.6 (3.2, 6.1)<br>Italy: 3.3 (1.5, 5.2)<br>Spain: 5.4 (4.4, 6.5)<br>Germany: 7.5 (−3.4,	**iCCA (males)**<br>**Asia:**<br>China: 10.7 (-2.7, 26.0)<br>Japan: −0.6 (-5.0, 4.1)<br>South Korea: 4.1 (3.1, 5.1)<br>Thailand: −0.8 (−4.2, 2.7)<br>**EU-5 and Nordics:**<br>Denmark: -2.0 (-6.9, 3.3)<br>Norway: 5.2 (2.4, 8.0)<br>France: 6.3 (−0.7, 13.9)<br>UK: 4.2 (2.2, 6.2)<br>Italy: 2.7 (−2.1, 7.6)<br>Spain: 6.3 (2.7, 10.0)<br>Germany: 6.3 (−5.1, 19.1)	**iCCA (females)**<br>**Asia:**<br>China: 11.7 (−6.2, 33.0)<br>Japan: −0.7 (−6.0, 4.8)<br>South Korea: 4.8 (3.2, 6.3)<br>Thailand: −1.3 (−3.1, 0.5)<br>**EU-5 and Nordics:**<br>Denmark: 0.1 (−3.8, 4.1)<br>Norway: 5.2 (−2.0, 12.8)<br>France: 6.3 (2.2, 10.5)<br>UK: 5.1 (3.9, 6.3)<br>Italy: 4.5 (2.0, 7.0)<br>Spain: 4.3 (2.7, 5.9)<br>Germany: 9.2 (−3.0, 22.9)	**eCCA (both sexes):**<br>**Asia:**<br>China: NR<br>Japan: 1.4 (−1.0, 3.8)<br>South Korea: 3.4 (−0.6, 7.7)<br>Thailand: 8.8 (−8.6, 29.6)<br>**EU-5 and Nordics:**<br>Denmark: NR<br>Norway: 2.4 (−4.6, 9.8)<br>France: 2.0 (0.2, 3.7)<br>UK: 3.3 (−1.9, 8.7)<br>Italy: 4.0 (−0.4, 8.6)<br>Spain: 2.4 (−0.5, 5.3)<br>Germany: 3.0 (−7.7, 15.0)<br>**US:**<br>US: 2.4	**eCCA (males)**<br>**Asia:**<br>China: NR<br>Japan: 1.7 (−0.5, 3.9)<br>South Korea: 3.5 (−1.8, 9.1)<br>Thailand: 9.4 (-12.4, 36.7)<br>**EU-5 and Nordics:**<br>Denmark: NR<br>Norway: 3.7 (−3.2, 11.1)<br>France: 2.8 (1.9, 3.7)<br>UK: 3.0 (−3.4, 9.7)<br>Italy: 3.8 (0.0, 7.7)<br>Spain: 3.4 (0.8, 6.0)<br>Germany: 3.3 (−5.5, 13.0)<br>**US:**<br>US: 2.5 (−0.1, 5.2)	**eCCA (females)**<br>**Asia:**<br>China: NR<br>Japan: 0.9 (−1.5, 3.2)<br>South Korea: 3.2 (0.4, 6.1)<br>Thailand: 7.9 (−4.5, 22.1)<br>**EU-5 and Nordics:**<br>Denmark: NR<br>Norway: 0.9 (−6.8, 9.2)<br>France: 1.0 (−3.2, 5.5)<br>UK: 3.5 (−3.4, 10.8)<br>Italy: 4.1 (−1.0, 9.5)<br>Spain: 0.7 (−4.1, 5.8)<br>Germany: 2.2 (−11.5, 18.0)<br>**US:**<br>US: 1.9 (−0.7, 4.5)

<table>
<tr><th>Study/database</th><th>Dataset/registry</th><th>Year(s)</th><th>Cancer site</th><th>Definition[a]</th><th colspan="6">Estimated APC in incidence, % (95% CI)</th></tr>
<tr><td></td><td></td><td></td><td></td><td></td><td>19.6)
<u>US:</u> 2.0 (−2.3, 6.5)</td><td><u>US:</u>
US: 1.0 (−4.0, 6.3)</td><td><u>US:</u>
US: 3.0 (−0.7, 6.8)</td><td>(0, 4.8)</td><td></td><td></td></tr>
<tr><td>GLOBOCAN [53]</td><td>Global data, GLOBOCAN</td><td>2008–2012 with the following exceptions:
Denmark, Norway and Sweden (2012–2016)
Japan (2006–2010)
UK (England and Wales; 2013–2017)
UK (N.Ireland; 2009–2013)</td><td>GBC</td><td>ICD-10 C23</td><td colspan="3">GBC (males)
<u>Asia:</u>
China: −2.67
Japan: 0.49
South Korea: 2.95
Thailand: −0.96
<u>EU-5 and Nordics:</u>
Denmark: −0.16
Norway: −5.54
Sweden: 0.07
France: 4.49
UK (England and Wales): −1.56
UK (N. Ireland): 13.10
Italy: 4.95
Spain: −0.92
Germany: 1.25
<u>US:</u>
US: −1.08</td><td colspan="3">GBC (females)
<u>Asia:</u>
China: −3.14
Japan: −1.62
South Korea: −0.53
Thailand: 8.92
<u>EU-5 and Nordics:</u>
Denmark: −3.30
Norway: −11.47
Sweden: −0.38
France: 2.93
UK (England and Wales): 1.12
UK (N. Ireland): −1.24
Italy: −5.70
Spain: −5.04
Germany: −2.80
<u>US:</u>
US: −1.25</td></tr>
<tr><td>SEER-22 [27]</td><td>US, SEER-22</td><td>2015-2019</td><td>GBC
C23.9</td><td>US, SEER-22</td><td colspan="6">Both sexes: −0.1 (−0.5, 0.5)
Males: −0.9 (−1.2, −0.6)
Females: 0.0 (−0.5, 0.7)</td></tr>
<tr><td>Ali 2022 [1]</td><td>US, SEER-18</td><td>2000-2017</td><td>iCCA</td><td>ICD-0-3/WHO 2008: iCCA C22.1</td><td colspan="6">Both sexes: 5.7 (4.3, 7.2); p<0.05
Males: 5.5; p<0.05
Females: 5.9; p<0.05</td></tr>
<tr><td>Koshiol 2022 [16]</td><td>US, NPCR-SEER database
SEER-18 (18 registries)</td><td>2001-2015</td><td>eCCA, iCCA, AC, GBC</td><td>ICD-O-3
eCCA C24.0
iCCA C22.1
GBC C23.9
AC C24.1</td><td colspan="3">BTC:
Both sexes:1.76
Males: 1.76
Females: 1.71
GBC:</td><td colspan="3">iCCA:
Both sexes: 6.65
Males: 6.24
Females: 7.00
eCCA:</td></tr>
</table>

Study/database	Dataset/registry	Year(s)	Cancer site	Definition[a]	Estimated APC in incidence, % (95% CI)	
				Klatskin tumours[b]	Both sexes: −0.25<br>Males: −0.09<br>Females: −0.21<br>**AC:**<br>Both sexes: −0.01<br>Males: −0.14<br>Females: −0.08	Both sexes: 0.72<br>Males: 0.53<br>Females: 0.77
Xing 2022 [32]	US, SEER	2000-2018	iCCA	C22.1	7.3% (6.2, 8.4)	
Javle 2022 [12]	US, SEER-18	2013-2017	CCA	ICD-O-3<br>iCCA: C22.1<br>eCCA: C24.0, C24.1, C24.9	**CCA:** 2.38 (0.93, 3.86)<br>**iCCA:** 9.26 (5.62, 13.02)<br>**eCCA:** −1.97 (−5.41, 1.6)	
Mancini 2022 [20]	Italy, 13 cancer registries	2003-2017	iCCA	ICD-O-3 C22	Males: 4.0% (2.7%, 5.3%)<br>Females: 3.6% (2.1%, 5.0%)	
Ghiringhelli 2023 [9]	France, Digestive Cancer Registry of Burgundy	2012-2019	BTC (iCCA, eCCA, GBC, AC)	ICD-O-3 C22.1 (iCCA), C23 (GBC), C24.0 (eCCA), C24.1 (AC)	**AC**<br>Males: −14.6% (−26.0, −3.3) p=0.012<br>Females: −16.6% (−28.4, −4.8) p=0.006	**iCCA**<br>Males: 6.8% (0.1, 13.6) p=0.010
Rahman 2022 [24]	Sweden, Swedish National Cancer Register	1993-2019	BTC, iCCA, GBC pCCA[b] and dCCA[b]	ICD-0-2<br>iCCA: C22.0/22.1; GBC: 23.9; pCCA[b] C24.0; dCCA[b] C24.1/C17.0	**BTC:** −0.13 (−0.34, 0.08), p=0.242<br>**GBC:** −2.82 (−3.18, −2.46), p=0.000	**iCCA:** 1.74 (1.30, 2.18), p=0.000<br>**pCCA:**[b] 3.62 (3.06, 4.19), p=0.000<br>**dCCA:**[b] 1.40 (0.88, 1.93), p=0.000
An 2023 [3]	National Central Cancer Registry, China	2006-2015	iCCA	ICD-0-3 22.0, 22.1	Both sexes: 2.1 (−0.9, 5.1); p>0.05<br>Males: 2.8 (−0.6, 6.2); p>0.05<br>Females: 1.0 (−1.6, 3.6); p>0.05	
Kang 2022 [14]	Korea, KCCR based on the Korea National Cancer Incidence Database	1999-2019	eCCA, GBC	ICD-10 GBC: C23; eCCA: C24	**GBC:**<br>Both sexes: −0.87 (−0.46, −0.29); p<0.01<br>Males: −1.23 (1.65, −0.8) p<0.01	**eCCA:**<br>Both sexes: 0.76 (0.52, 1.0); p<0.01<br>Males: 0.63 (0.37, 0.90); p<0.01<br>Females: 0.64 (0.31, 0.96);

Study/database	Dataset/registry	Year(s)	Cancer site	Definition[a]	Estimated APC in incidence, % (95% CI)	
					Females: −0.75 (−1.41, −0.08) p=0.03	p<0.01
Hong 2022 [11]	Korea, KCCR based on the Korea National Cancer Incidence Database	2008-2019	iCCA	ICD-10 C22.1	−4.38; p<0.01	
Cao 2022 [6]	Thailand (Lampang)	1993-2012	CCA	ICD-O-3:<br>CCA: C22.1, C24.0, C24.8 or C24.9	Males: 5.0 (3.7, 6.3); p<0.05<br>Females: 2.02 (0.9, 3.2); p<0.05	
Kamsa-Ard 2021 [13]	Thailand, KKCR, Northeast Thailand	1989-2018	CCA	ICD-O-3: C22.1, C24.0, C24.8, C24.9 (excluded C24.1 - Ampulla of Vater)	-2.9% (-3.8, -2.0); p<0.001	

AC, ampullary cancer; APC, annual percentage change; BTC, biliary tract cancer; CCA, cholangiocarcinoma; dCCA, distal cholangiocarcinoma; eCCA, extrahepatic cholangiocarcinoma; GBC, gallbladder cancer; GBD, Global Burden of Disease; iCCA, intrahepatic cholangiocarcinoma; ICD (O), International Classification of Diseases (Oncology); KCCR, Korea Central Cancer Registry; KKCR, Khon Kaen Cancer Registry; NPCR, National Program of Cancer Registries; pCCA, perihilar cholangiocarcinoma; SEER, Surveillance, Epidemiology, and End Results; UK, United Kingdom; US, United States; WHO, World Health Organization

[a] ICD-O-3 morphology codes for each study are provided in Table S1. [b] dCCA and pCCA, also called hCCA or 'Klatskin tumours', are subtypes of eCCA.

Table S8: Global studies: Prevalence of BTC

Study/Database	Dataset/Registry	Year(s)	Cancer site/definition[a]	5-year prevalence	1-year prevalence, cases (rate/100, 000)
GLOBOCAN [10]	Global data, GLOBOCAN	2020	GBC<br>C23	**US**<br>Both sexes: 5, 659 (1.7/100, 000)<br>Males: 2, 136 (1.3/100, 000)<br>Females: 3, 523 (2.1/100, 000)<br>**France**<br>Both sexes: 721 (1.1/100, 000)<br>Males: 288 (0.91/100, 000)<br>Females: 433 (1.3/100, 000)<br>**Germany**<br>Both sexes: 1, 543 (1.8/100, 000)<br>Males: 548 (1.3/100, 000)<br>Females: 995 (2.3/100, 000)<br>**Italy**<br>Both sexes: 1, 148 (1.9/100, 000)<br>Males: 477 (1.6/100, 000)<br>Females: 671 (2.2/100, 000)<br>**Spain**<br>Both sexes: 736 (1.6/100, 000)<br>Males: 309 (1.3/100, 000)<br>Females: 427 (1.8/100, 000)<br>**UK**<br>Both sexes: 1, 382 (2.0/100, 000)<br>Males: 415 (1.2/100, 000)<br>Females: 967 (2.8/100, 000)<br>**Denmark**<br>Both sexes: 73 (1.3/100, 000)<br>Males: 32 (1.1/100, 000)	**US**<br>Both sexes: 2, 591 (0.78/100, 000)<br>Males: 950 (0.58/100, 000)<br>Females: 1, 641 (0.98/100, 000)<br>**France**<br>Both sexes: 332 (0.51/100, 000)<br>Males: 130 (0.41/100, 000)<br>Females: 202 (0.6/100, 000)<br>**Germany**<br>Both sexes: 716 (0.85/100, 000)<br>Males: 245 (0.59/100, 000)<br>Females: 471 (1.1/100, 000)<br>**Italy**<br>Both sexes: 530 (0.88/100, 000)<br>Males: 213 (0.72/100, 000)<br>Females: 317 (1/100, 000)<br>**Spain**<br>Both sexes: 343 (0.73/100, 000)<br>Males: 139 (0.6/100, 000)<br>Females: 204 (0.86/100, 000)<br>**UK**<br>Both sexes: 638 (0.94/100, 000)<br>Males: 185 (0.55/100, 000)<br>Females: 453 (1.3/100, 000)<br>**Denmark**

Study/Database	Dataset/Registry	Year(s)	Cancer site/definition[a]	5-year prevalence	1-year prevalence, cases (rate/100, 000)
				Females: 41 (1.4/100, 000)<br>**Finland**<br>Both sexes: 161 (2.9/100, 000)<br>Males: 70 (2.6/100, 000)<br>Females: 91 (3.2/100, 000)<br>**Norway**<br>Both sexes: 59 (1.1/100, 000)<br>Males: 27 (1.0/100, 000)<br>Females: 32 (1.2/100, 000)<br>**Sweden**<br>Both sexes: 268 (2.7/100, 000)<br>Males: 129 (2.5/100, 000)<br>Females: 139 (2.8/100, 000)<br>**China**<br>Both sexes: 33, 640 (2.3/100, 000)<br>Males: 13, 966 (1.9/100, 000)<br>Females: 19, 674 (2.8/100, 000)<br>**Japan**<br>Both sexes: 9, 744 (7.7/100, 000)<br>Males: 4, 541 (7.4/100, 000)<br>Females: 5, 203 (8.0/100, 000)<br>**South Korea**<br>Both sexes: 4, 110 (8.0/100, 000)<br>Males: 2, 074 (8.1/100, 000)<br>Females: 2, 036 (8.0/100, 000)<br>**Thailand**<br>Both sexes: 2, 169 (3.1/100, 000)<br>Males: 1, 218 (3.6/100, 000)<br>Females: 951 (2.7/100, 000)	Both sexes: 33 (0.57/100, 000)<br>Males: 14 (0.49/100, 000)<br>Females: 19 (0.65/100, 000)<br>**Finland**<br>Both sexes: 75 (1.4/100, 000)<br>Males: 32 (1.2/100, 000)<br>Females: 43 (1.5/100, 000)<br>**Norway**<br>Both sexes: 28 (0.52/100, 000)<br>Males: 12 (0.44/100, 000)<br>Females: 16 (0.6/100, 000)<br>**Sweden**<br>Both sexes: 122 (1.2/100, 000)<br>Males: 57 (1.1/100, 000)<br>Females: 65 (1.3/100, 000)<br>**China**<br>Both sexes: 15, 298 (1.1/100, 000)<br>Males: 6, 177 (0.83/100, 000)<br>Females: 9, 121 (1.3/100, 000)<br>**Japan**<br>Both sexes: 4, 537 (3.6/100, 000)<br>Males: 2, 039 (3.3/100, 000)<br>Females: 2, 498 (3.9/100, 000)<br>**South Korea**<br>Both sexes: 1, 871 (3.6/100, 000)<br>Males: 921 (3.6/100, 000)<br>Females: 950 (3.7/100, 000)<br>**Thailand**<br>Both sexes: 984 (1.4/100, 000)<br>Males: 541 (1.6/100, 000)

Study/Database	Dataset/Registry	Year(s)	Cancer site/definition[a]	5-year prevalence	1-year prevalence, cases (rate/100, 000)
					Females: 443 (1.2/100, 000)

BTC, biliary tract cancer; GBC, gallbladder cancer; GLOBOCAN, Global Cancer Observatory; UK, United Kingdom; US, United States

[a] ICD-O-3 morphology codes for each study are provided in Table S1.

Table S9: United States: Prevalence of BTC

US Study/Database	Dataset/Registry	Year(s)	Cancer site/definition[a]	5-year prevalence	1-year prevalence
SEER [27]	US, SEER-12	2020	Liver and intrahepatic bile duct cancer<br>C220, C221	**Complete prevalence**<br>Both sexes: 105, 765<br>Males: 72, 118<br>Females: 33, 647	
SEER [27]	US, SEER-12	2020	GBC<br>C23.9	**Complete prevalence**<br>Both sexes: 13, 630<br>Males: 3, 410<br>Females: 10, 220	
Alkhayyat 2021 [2]	US, 'Explorys' EHR data (26 health systems across the US)	1999-2019	GBC<br>SNOMED-CT:<br>'Primary malignant neoplasm of the bladder'	**20-yr prevalence/100, 000:**<br>Both sexes: 8.5<br>Females: 9.7<br>Males: 7.0	NR
Javle 2022 [12]	US, SEER-18	2007-2012	CCA, eCCA, iCCA<br>iCCA: C22.1<br>eCCA: C24.0, C24.1, C24.9	Prevalence/100, 000:<br>CCA: 3.93<br>iCCA: 0.98<br>eCCA: 2.96	NR

US Study/Database	Dataset/Registry	Year(s)	Cancer site/definition[a]	5-year prevalence	1-year prevalence
Koshiol 2022 [16]	US, NPCR-SEER database,<br>SEER-18 (18 registries)	2005-2015	BTC<br>eCCA: C24.0 (incl. Klatskin tumours[b])<br>iCCA: C22.1; C22.0<br>GBC: C23.9<br>AC: C24.1<br>Klatskin tumours[b]	**10-yr prevalence rate**<br>BTC: 10.8<br>GBC: 3.5<br>iCCA: 2.3<br>eCCA: 2.0<br>AC: 2.8	NR

AC, ampullary cancer; BTC, biliary tract cancer; CCA, cholangiocarcinoma; eCCA, extrahepatic cholangiocarcinoma; EHR, electronic health record; GBC, gallbladder cancer; iCCA, intrahepatic cholangiocarcinoma; ICD (O), International Classification of Diseases (Oncology); NPCR, National Program of Cancer Registries; NR, not reported; SEER, Surveillance, Epidemiology, and End Results; SNOMED-CT, Systematized Nomenclature of Medicine-Clinical Terms UK, United Kingdom; US, United States; yr, year

[a] ICD-O-3 morphology codes for each study are provided in Table S1. [b] Klatskin tumours, also called hCCA or pCCA, are subtypes of eCCA.

Table S10:Asia: Prevalence of BTCs

Database/Study	Dataset/registry	Year(s)	Cancer site, definition	5-year prevalence	1-year prevalence (rate/100, 000)
Chen 2022 [7]	Mainland China, Global Burden of Disease Study, 2019	2019	BTC+GBC	NR	Both sexes: 47, 278 (2.40/100, 000)<br>Males: 24, 133 (15, 812, 30, 655)<br>Females: 23, 145 (13, 575, 30, 377)

BTC, biliary tract cancer; GBC, gallbladder cancer; NR, not reported

Table S11. Search terms used to retrieve articles on the epidemiology of BTC

Search Number	Search Terms
1	*Biliary Tract Neoplasms/ or *bile duct tumor/
2	(((biliary tract or ampullary or gallbladder or bile duct) adj2 (cancer$ or neoplasm$ or carcinoma or adenocarcinoma or tum?r)) or cholangiocarcinoma).ti, ab.
3	1 or 2
4	exp *epidemiology/ or exp *incidence/ or exp *prevalence/ or (epidemiolog$ or incidence or prevalence).ti, ab.
5	3 and 4
6	exp Longitudinal Studies/ or exp Retrospective Studies/ or exp Prospective Studies/ or exp Cohort Studies/ or exp Cross-Sectional Studies/ or exp Observational Study/ or (longitudinal study or retrospective study or prospective study or cohort$ or follow up or cross-sectional study or cross sectional study or followup study or observational study or registry or registries or real world or cross sectional or claims database or electronic health record$ or EHR or electronic medical record$ or EMR$ or RWE).ti, ab.
7	5 and 6
8	7 not (animals/ not humans/)
9	8 not ((exp animal/ or nonhuman/) not exp human/)
10	case report/ or case reports/ or case study/ or case report$.jx. or case report$.jw. or (case report or case study or case series or woman or man or child or adolescent or female or male or boy or girl or infant or unusual case).ti.
11	(Ephemera or "Introductory Journal Article" or News or "Newspaper Article" or Editorial or Comment or Overall or Letter or Short Survey or Tombstone or Books).pt. or in vitro Techniques/ or in vitro study/ or (in vitro or commentary or editorial or comment or letter or mice or rat or mouse or animal or murine).ti.
12	review.pt. not (systematic or (meta and analy*) or ((indirect or mixed) and treatment comparison)).ti, ab.
13	9 not (10 or 11 or 12)
14	limit 13 to (article or article in press)
15	13 and (asco or American Society of Clinical Oncology or esmo or European Society of Medical Oncology or Japanese Society of Medical Oncology).cf, cg, nc.
16	14 or 15
17	remove duplicates from 16
18[a]	limit 17 to yr="2020-Current"

BTC, biliary tract cancer

[a] The search was later extended to capture articles published between 13 March 2023 and 11 September 2023

Table S12. Search terms to retrieve articles investigating MDM2 in BTC

Search Number	Search Terms
1	Exp Biliary Tract Neoplasms/ or exp bile duct tumor/
2	(((biliary tract or ampullary or gallbladder or bile duct) adj4 (cancer$ or neoplasm$ or carcinoma or adenocarcinoma or tum?r)) or cholangiocarcinoma).ti, ab.
3	1 or 2
4	Exp Proto-Oncogene Proteins c-mdm2/ or exp mouse double minute 2 homolog/ or (MDM2 or MDM-2 or Proto-Oncogene Proteins c-mdm2 or Proto-Oncogene Protein c-mdm2 or mouse double minute 2 homolog).ti, ab.
5	3 and 4
6	5 not (animals/ not humans/)
7	6 not ((exp animal/ or nonhuman/) not exp human/)
8	limit 7 to (article or article in press)
9	7 and (asco or American Society of Clinical Oncology or esmo or European Society of Medical Oncology or Japanese Society of Medical Oncology).cf, cg, nc.
10	8 or 9
11	remove duplicates from 10

BTC, biliary tract cancer; MDM2, mouse double minute 2 homolog

Supplemental References

1. Ali, H., B. Tedder, S.H. Waqar, R. Mohamed, E.L. Cate, and E. Ali. Changing incidence and survival of intrahepatic cholangiocarcinoma based on Surveillance, Epidemiology, and End Results Database (2000-2017). *Ann Hepatobiliary Pancreat Surg*. 2022;26(3):235-243. DOI: 10.14701/ahbps.21-173.
2. Alkhayyat, M., M. Abou Saleh, T. Qapaja, M. Abureesh, A. Almomani, E. Mansoor, and P. Chahal. Epidemiology of gallbladder cancer in the Unites States: a population-based study. *Chin Clin Oncol*. 2021;10(3):25. DOI: 10.21037/cco-20-230.
3. An, L., R. Zheng, S. Zhang, R. Chen, S. Wang, K. Sun, et al. Hepatocellular carcinoma and intrahepatic cholangiocarcinoma incidence between 2006 and 2015 in China: estimates based on data from 188 population-based cancer registries. *Hepatobiliary Surg Nutr*. 2023;12(1):45-55. DOI: 10.21037/hbsn-21-75.
4. Baria, K., E. De Toni, B. Yu, Z. Jiang, S. Kabadi, and M. Malvezz. Worldwide Incidence and Mortality of Biliary Tract Cancer. *Gastro Hep Advances*. 2022;1(4):618-626.
5. Barner-Rasmussen, N., E. Pukkala, K. Hadkhale, and M. Farkkila. Risk factors, epidemiology and prognosis of cholangiocarcinoma in Finland. *United European Gastroenterol J*. 2021;9(10):1128-1135. DOI: 10.1002/ueg2.12154.
6. Cao, P., L.S. Rozek, D. Pongnikorn, H. Sriplung, and R. Meza. Comparison of Cholangiocarcinoma and Hepatocellular Carcinoma Incidence Trends from 1993 to 2012 in Lampang, Thailand. *Int J Environ Res Public Health*. 2022;19(15) DOI: 10.3390/ijerph19159551.
7. Chen, S., K. Han, Y. Song, S. Liu, X. Li, S. Wang, et al. Current status, trends, and predictions in the burden of gallbladder and biliary tract cancer in China from 1990 to 2019. *Chin Med J (Engl)*. 2022;135(14):1697-1706. DOI: 10.1097/CM9.0000000000002258.
8. Florio, A.A., J. Ferlay, A. Znaor, D. Ruggieri, C.S. Alvarez, M. Laversanne, et al. Global trends in intrahepatic and extrahepatic cholangiocarcinoma incidence from 1993 to 2012. *Cancer*. 2020;126(11):2666-2678. DOI: 10.1002/cncr.32803.
9. Ghiringhelli, F., V. Jooste, S. Manfredi, A. Hennequin, C. Lepage, and A.M. Bouvier. Biliary tract cancers have distinct epidemiological patterns and clinical characteristics according to tumour site. *HPB (Oxford)*. 2023 DOI: 10.1016/j.hpb.2023.02.016.
10. Ferlay, J., M. Ervik, F. Lam, M. Laversanne, M. Colombet, L. Mery, et al., *Global Cancer Observatory: Cancer Today. Lyon, France: International Agency for Research on Cancer.* https://gco.iarc.who.int/today. *Accessed 30 March*. 2023.
11. Hong, S.Y., M.J. Kang, T. Kim, K.W. Jung, and B.W. Kim. Incidence, mortality, and survival of liver cancer using Korea central cancer registry database: 1999-2019. *Ann Hepatobiliary Pancreat Surg*. 2022;26(3):211-219. DOI: 10.14701/ahbps.22-044.
12. Javle, M., S. Lee, N.S. Azad, M.J. Borad, R. Kate Kelley, S. Sivaraman, et al. Temporal Changes in Cholangiocarcinoma Incidence and Mortality in the United States from 2001 to 2017. *Oncologist*. 2022;27(10):874-883. DOI: 10.1093/oncolo/oyac150.
13. Kamsa-Ard, S., C. Santong, S. Kamsa-Ard, V. Luvira, V. Luvira, K. Suwanrungruang, and V. Bhudhisawasdi. Decreasing trends in cholangiocarcinoma incidence and relative survival in Khon Kaen, Thailand: An updated, inclusive, population-based cancer registry analysis for 1989-2018. *PLoS One*. 2021;16(2):e0246490. DOI: 10.1371/journal.pone.0246490.

14. Kang, M.J., E.H. Yun, K.W. Jung, and S.J. Park. Incidence, mortality and survival of gallbladder, extrahepatic bile duct, and pancreatic cancer using Korea central cancer registry database: 1999-2019. *Ann Hepatobiliary Pancreat Surg*. 2022;26(3):220-228. DOI: 10.14701/ahbps.22-041.
15. Koppatz, H., S. Takala, K. Peltola, A. But, H. Makisalo, A. Nordin, and V. Sallinen. Gallbladder cancer epidemiology, treatment and survival in Southern Finland - a population-based study. *Scand J Gastroenterol*. 2021;56(8):929-939. DOI: 10.1080/00365521.2021.1915373.
16. Koshiol, J., B. Yu, S.M. Kabadi, K. Baria, and R.T. Shroff. Epidemiologic patterns of biliary tract cancer in the United States: 2001-2015. *BMC Cancer*. 2022;22(1):1178. DOI: 10.1186/s12885-022-10286-z.
17. Lee, Y.T., J.J. Wang, M. Luu, M. Noureddin, N.N. Nissen, T.C. Patel, et al. Comparison of Clinical Features and Outcomes Between Intrahepatic Cholangiocarcinoma and Hepatocellular Carcinoma in the United States. *Hepatology*. 2021;74(5):2622-2632. DOI: 10.1002/hep.32007.
18. Lin, C.R., Y.K. Lee, C.J. Chiang, Y.W. Yang, H.C. Chang, and S.L. You. Secular trends of intrahepatic cholangiocarcinoma in a high endemic area: A population-based study. *World J Gastroenterol*. 2022;28(28):3695-3705. DOI: 10.3748/wjg.v28.i28.3695.
19. Makiuchi, T. and T. Sobue. Descriptive epidemiology of biliary tract cancer incidence and geographic variation in Japan. *Eur J Cancer Prev*. 2023;32(1):2-9. DOI: 10.1097/CEJ.0000000000000758.
20. Mancini, S., L. Bucchi, F. Zamagni, S. Guzzinati, L. Dal Maso, M. Rugge, et al. Trends in Liver Cancer Incidence and Survival in Italy by Histologic Type, 2003-2017. *Cancers (Basel)*. 2022;14(24) DOI: 10.3390/cancers14246162.
21. Miranda-Filho, A., M. Pineros, C. Ferreccio, V. Adsay, I. Soerjomataram, F. Bray, and J. Koshiol. Gallbladder and extrahepatic bile duct cancers in the Americas: Incidence and mortality patterns and trends. *Int J Cancer*. 2020;147(4):978-989. DOI: 10.1002/ijc.32863.
22. Neuzillet, C., C. Emery, C. Teissier, S. Bouee, and A. Lievre. Patient healthcare trajectories of intrahepatic cholangiocarcinoma in France: A nationwide retrospective analysis. *Lancet Reg Health Eur*. 2022;15:100324. DOI: 10.1016/j.lanepe.2022.100324.
23. Rahib, L., M.R. Wehner, L.M. Matrisian, and K.T. Nead. Estimated Projection of US Cancer Incidence and Death to 2040. *JAMA Netw Open*. 2021;4(4):e214708. DOI: 10.1001/jamanetworkopen.2021.4708.
24. Rahman, R., J.F. Ludvigsson, E. von Seth, J. Lagergren, A. Bergquist, and C. Radkiewicz. Age trends in biliary tract cancer incidence by anatomical subtype: A Swedish cohort study. *Eur J Cancer*. 2022;175:291-298. DOI: 10.1016/j.ejca.2022.08.032.
25. Raza, S.A., W.L. da Costa, and A.P. Thrift. Increasing Incidence of Gallbladder Cancer among Non-Hispanic Blacks in the United States: A Birth Cohort Phenomenon. *Cancer Epidemiol Biomarkers Prev*. 2022;31(7):1410-1417. DOI: 10.1158/1055-9965.EPI-21-1452.
26. Rumgay, H., J. Ferlay, C. de Martel, D. Georges, A.S. Ibrahim, R. Zheng, et al. Global, regional and national burden of primary liver cancer by subtype. *Eur J Cancer*. 2022;161:108-118. DOI: 10.1016/j.ejca.2021.11.023.
27. SEER*Explorer, *An interactive website for SEER cancer statistics. Surveillance Research Program, National Cancer Institute.* https://seer.cancer.gov/statistics-network/explorer/. *Accessed January* 2024.

28. Selvadurai, S., K. Mann, S. Mithra, J. Bridgewater, H. Malik, and S.A. Khan. Cholangiocarcinoma miscoding in hepatobiliary centres. *Eur J Surg Oncol*. 2021;47(3 Pt B):635-639. DOI: 10.1016/j.ejso.2020.09.039.
29. Tella, S.H., M. Wieczorek, D. Hodge, and A. Mahipal. A glimpse into the future of cholangiocarcinoma: Predicting the future incidence based on the current epidemiological data. *Journal of Clinical Oncology*. 2023;41(4_suppl):616-616. DOI: 10.1200/JCO.2023.41.4_suppl.616.
30. Velasco, A.G., M. Quintana, M.P. Guinart, W. Carbajal, R.G. Sanchez, V. Anna, et al. P-216 Incidence and trends of biliary tract cancer in Girona: A population-based study from the Girona Cancer Registry (1994-2016). *Annals of Oncology*. 2020;31:S160-S161. DOI: 10.1016/j.annonc.2020.04.298.
31. Xie, W., T. Yang, J. Zuo, Z. Ma, W. Yu, Z. Hu, and Z. Song. Chinese and Global Burdens of Gastrointestinal Cancers From 1990 to 2019. *Front Public Health*. 2022;10:941284. DOI: 10.3389/fpubh.2022.941284.
32. Xing, H., B. Tan, C. Yang, and M. Zhang. Incidence Trend and Competing Risk Analysis of Patients With Intrahepatic Cholangiocarcinoma: A Population-Based Study. *Front Med (Lausanne)*. 2022;9:846276. DOI: 10.3389/fmed.2022.846276.
33. Zhu, M.X. and Y. Li. The correlations between socioeconomic status and intrahepatic cholangiocarcinoma in the United States: a population-based study. *Transl Cancer Res*. 2020;9(8):4931-4942. DOI: 10.21037/tcr-20-2506.
34. Battaglin, F., J. Xiu, Y. Baca, A.F. Shields, R.M. Goldberg, A. Puccini, et al. Comprehensive profiling of MDM2 amplified gastrointestinal (GI) cancers. *Annals of Oncology*. 2020;31(Supplement 4):S1100. DOI: 10.1016/j.annonc.2020.08.1344.
35. Bouattour, M., W. Juan, J.W. Valle, A. Vogel, J.W. Kim, M. Kitano, et al. *Characterization of long-term survivors in the TOPAZ-1 study of durvalumab or placebo plus gemcitabine and cisplatin in advanced biliary tract cancer.* in *ASCO Gastrointestinal Cancers Symposium*. 2023. San Francisco, CA
36. Cassier, P., C. De La Fouchardiere, P. Guibert, D. Pissaloux, C. Pacaux, C. Terret, et al. Actionable molecular alterations in advanced biliary tract carcinomas: Preliminary data from the ProfiLER program (NCT01774409). *Annals of Oncology*. 2017;28(Supplement 5):v247. DOI: 10.1093/annonc/mdx369.109.
37. D'Afonseca, V., A.D. Arencibia, A. Echeverria-Vega, L. Cerpa, J.P. Cayun, N.M. Varela, et al. Identification of Altered Genes in Gallbladder Cancer as Potential Driver Mutations for Diagnostic and Prognostic Purposes: A Computational Approach. *Cancer Informatics [Electronic Resource]*. 2020;19:1176935120922154. DOI: 10.1177/1176935120922154.
38. Feng, F., X. Wu, X. Shi, Q. Gao, Y. Wu, Y. Yu, et al. Comprehensive analysis of genomic alterations of Chinese hilar cholangiocarcinoma patients. *International Journal of Clinical Oncology*. 2021;26(4):717-727. DOI: 10.1007/s10147-020-01846-z.
39. Harthimmer, M.R., U. Stolborg, P. Pfeiffer, M.B. Mortensen, C. Fristrup, and S. Detlefsen. Mutational profiling and immunohistochemical analysis of a surgical series of ampullary carcinomas. *Journal of Clinical Pathology*. 2019;72(11):762-770. DOI: 10.1136/jclinpath-2019-205912.
40. Kato, S., J.S. Ross, L. Gay, F. Dayyani, J. Roszik, V. Subbiah, and R. Kurzrock. Analysis of MDM2 Amplification: Next-Generation Sequencing of Patients With Diverse Malignancies. *JCO Precis Oncol*. 2018;2018 DOI: 10.1200/PO.17.00235.
41. Kendre, G., K. Murugesan, T. Brummer, O. Segatto, A. Saborowski, and A. Vogel. Charting co-mutation patterns associated with actionable drivers in intrahepatic

cholangiocarcinoma. *Journal of Hepatology*. 2023;78(3):614-626. DOI: 10.1016/j.jhep.2022.11.030.
42. Kim, S.J., M. Akita, Y.N. Sung, K. Fujikura, J.H. Lee, S. Hwang, et al. MDM2 Amplification in Intrahepatic Cholangiocarcinomas: Its Relationship With Large-Duct Type Morphology and Uncommon KRAS Mutations. *American Journal of Surgical Pathology*. 2018;42(4):512-521. DOI: 10.1097/PAS.0000000000001006.
43. Kumar-Sinha, C., P. Vats, N. Tran, D.R. Robinson, V. Gunchick, Y.M. Wu, et al. Genomics driven precision oncology in advanced biliary tract cancer improves survival. *Neoplasia*. 2023;42:100910. DOI: 10.1016/j.neo.2023.100910.
44. Lee, H., K. Wang, A. Johnson, D.M. Jones, S.M. Ali, J.A. Elvin, et al. Comprehensive genomic profiling of extrahepatic cholangiocarcinoma reveals a long tail of therapeutic targets. *Journal of Clinical Pathology*. 2016;69(5):403-8. DOI: 10.1136/jclinpath-2015-203394.
45. Lin, J., X. Yang, Y. Cao, G. Li, S. Zhao, J. Shi, et al. Genomics and translational precision oncology for 803 patients with biliary tract cancer. *Journal of Clinical Oncology. Conference*. 2020;38(15) DOI: 10.1200/JCO.2020.38.15_suppl.4589.
46. Lin, E.S., E.G. Mehlhaff, C.P. Bergstrom, D.M. Lesnik, N.K. LoConte, S.J. Lubner, et al. Actionable molecular alterations in veterans with advanced cholangiocarcinoma. *Journal of Clinical Oncology*. 2024;42:548.
47. MyCancerGenome, *Biomarkers- MDM2 Amplification.* https://www.mycancergenome.org/content/alteration/mdm2-amplification/. *Accessed 30 March*. 2023.
48. Pu, X., L. Zhu, F. Li, J. Zheng, H. Wu, Y. Fu, et al. Target molecular treatment markers in Intrahepatic Cholangiocarcinoma based on Chinese population. *Pathology, Research & Practice*. 2020;216(9):153116. DOI: 10.1016/j.prp.2020.153116.
49. Rimini, M., M. Rizzato, L. Rimassa, M. Niger, L. Fornaro, L. Antonuzzo, et al. P-343 The impact of genomic alterations on response rate and survival outcomes in advanced BTC patients who receive cisplatin/gemcitabine plus durvalumab in clinical practice. *Annals of Oncology* 2023;24.
50. Simbolo, M., A. Mafficini, C. Vicentini, A. Ruzzenente, B. Rusev, M. Brunelli, et al. Perihilar and distal extrahepatic cholangiocarcinomas show different genetic profiles but share MYC copy gain and TP53 mutation as independent poor prognostic markers. *Annals of Oncology*. 2019;30(Supplement 4):AA50. DOI: 10.1093/annonc/mdz183.
51. Wong, W., M.A. Lowery, M.F. Berger, Y. Kemel, B. Taylor, A. Zehir, et al. Ampullary cancer: Evaluation of somatic and germline genetic alterations and association with clinical outcomes. *Cancer*. 2019;125(9):1441-1448. DOI: 10.1002/cncr.31951.
52. Zheng, Y., Y. Qin, W. Gong, H. Li, B. Li, Y. Wang, et al. Specific genomic alterations and prognostic analysis of perihilar cholangiocarcinoma and distal cholangiocarcinoma. *Journal of Gastrointestinal Oncology*. 2021;12(6):2631-2642. DOI: 10.21037/jgo-21-776.
53. Ervik, M., F. Lam, M. Laversanne, J. Ferlay, and F. Bray, *Global Cancer Observatory: Cancer Over Time. Lyon, France: International Agency for Research on Cancer.* https://gco.iarc.fr/overtime. *Accessed 30th March*. 2023.
